# p38β - MAPK11 and its role in female cancers

**DOI:** 10.1186/s13048-021-00834-9

**Published:** 2021-06-26

**Authors:** Periklis Katopodis, Rachel Kerslake, Athanasios Zikopoulos, Nefeli Beri, Vladimir Anikin

**Affiliations:** 1 Biosciences, College of Health and Life Sciences, Brunel University London, Uxbridge, UK; 2 Division of Thoracic Surgery, The Royal Brompton & Harefield NHS Foundation Trust, Harefield Hospital, London, UB9 6JH UK; 3 Obstetrics and Gynaecology Department, Royal Cornwall Hospitals NHS Foundation Trust, Royal Cornwall Hospital, Truro, TR1 3LJ UK; 4 Department of Medicine, Karolinska Institutet, 17177 Stockholm, Sweden; 5 Department of Oncology and Reconstructive Surgery, Sechenov First Moscow State Medical University, Moscow, Russian Federation 119146

**Keywords:** MAPK11, MAPK, Female cancers, Breast cancer, Ovarian cancer, Cervical cancer, Uterine carcinosarcoma, Meexress, Smartapp, Cbioportal, canSar, Methylation, p38β, Pan-cancer

## Abstract

**Background:**

The p38MAPK family of Mitogen Activated Protein Kinases are a group of signalling molecules involved in cell growth, survival, proliferation and differentiation. The widely studied p38α isoform is ubiquitously expressed and is implicated in a number of cancer pathologies, as are p38γ and p38δ. However, the mechanistic role of the isoform, p38β, remains fairly elusive. Recent studies suggest a possible role of p38β in both breast and endometrial cancer with research suggesting involvement in bone metastasis and cancer cell survival. Female tissue specific cancers such as breast, endometrial, uterine and ovary account for over 3,000,000 cancer related incidents annually; advancements in therapeutics and treatment however require a deeper understanding of the molecular aetiology associated with these diseases. This study provides an overview of the MAPK signalling molecule p38β (MAPK11) in female cancers using an *in-silico* approach.

**Methods:**

A detailed gene expression and methylation analysis was performed using datasets from cBioportal, CanSar and MEXPRESS. Breast, Uterine Endometrial, Cervical, Ovarian and Uterine Carcinosarcoma TCGA cancer datasets were used and analysed.

**Results:**

Data using cBioportal and CanSAR suggest that expression of p38β is lower in cancers: BRCA, UCEC, UCS, CESC and OV compared to normal tissue. Methylation data from SMART and MEXPRESS indicate significant probe level variation of CpG island methylation status of the gene MAPK11. Analysis of the genes’ two CpG islands shows that the gene was hypermethylated in the CpG1 with increased methylation seen in BRCA, CESC and UCEC cancer data sets with a slight increase of expression recorded in cancer samples. CpG2 exhibited hypomethylation with no significant difference between samples and high levels of expression. Further analysis from MEXPRESS revealed no significance between probe methylation and altered levels of expression. In addition, no difference in the expression of BRCA oestrogen/progesterone/HER2 status was seen.

**Conclusion:**

This data provides an overview of the expression of p38β in female tissue specific cancers, showing a decrease in expression of the gene in BRCA, UCEC, CESC, UCS and OV, increasing the understanding of p38β MAPK expression and offering insight for future in-vitro investigation and therapeutic application.

**Supplementary Information:**

The online version contains supplementary material available at 10.1186/s13048-021-00834-9.

## Introduction

The establishment of cancer as one of the leading causes of morbidity and mortality globally is widely accepted, yet the complexity of molecular processes that contribute to the development and progression of the numerous subsets of cancer continues to plague researchers. Of the 18.1 million incidences of cancer worldwide in 2018 over 20% of cases are thought to be female reproductive tissue specific [1]. The most frequently diagnosed type of female specific cancers is breast cancer with 2,088,849 cases recorded in 2018, around only 1% of which are male [2]. Cervical cancer is the most frequently diagnosed of the gynaecological cancers, with 569,847 cases diagnosed annually followed by endometrial and uterine with 382,069 cases; ovarian is the least common yet deadliest with 295,414 cases diagnosed globally each year and over 184,779 deaths [1].

Knowledge regarding associated risk factors such as diet, weight, heritability, and menopausal status along with global health initiatives, routine cervical screening, and breast imaging, have led to earlier detection of both cervical and breast cancer [3, 4]. However, recurrence and mortality rates are still troublesome. Gynaecological cancers such as uterine and endometrial are also often detected at early to mid-stages with treatment for female reproductive tissue cancers often involving surgical procedures such as complete or partial mastectomy (breast) or hysterectomy [5]. Ovarian cancer, however, is more frequently diagnosed at an advanced stage owing to the ambiguous nature of symptoms related to many gynaecological disorders [6]. Despite comprising the lowest number of gynaecological cases, ovarian cancer is the deadliest of gynaecological malignancies with five-year survival less than 26% for those diagnosed with advanced serous ovarian cancer [7]. Notwithstanding the continued advancements in detection and treatments of cancer many of the molecular mechanisms implicated in the development, progression and resistance related recurrence of female reproductive tissue specific cancers require further investigation [8].

Of the multitude of risk factors associated with the development of cancer, hormone dysregulation is substantial in breast, with the onset of menopause lowering the risk of breast cancer while prolonged exposure to endogenous hormones such as oestrogen as and oestrogen receptor status presenting an increased risk [9]. In addition, age, as well as genome instability and mutation are often implicated in the development and progression of cancer. Heritable mutation of the DNA repair genes BRCA1 and BRCA2 are not only significantly associated with breast cancer but are also biomarkers of ovarian and endometrial cancer [10]. Other notable genes include the Tumour Suppressor protein 53 (TP53) as well as angiogenic factors such as VEGF and TGF-B as well as signalling molecules such as the Mitogen Activated Protein Kinase (MAPK) family kinase proteins [11].

### MAPKs

**Fig. 1:**
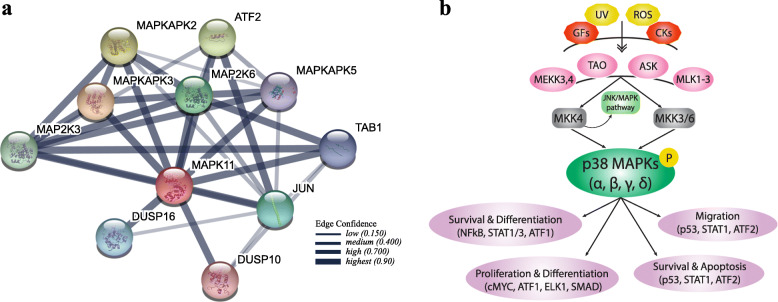
**a** MAPK11 protein association network acquired by StringDB; **b** MAPK signalling pathways showing the initiation of JNK and p38 following external stimulation from UV and Reactive Oxygen Species (yellow) as well as CKs and growth factors (red). The p38 isoforms (green) are phosphorylated via MKK3/6 and MKK4 (grey) in response to ROS and environmental stress consequently increasing activation of downstream targets involved in processes such as cell migration, survival and differentiation, proliferation and apoptosis. UV: Ultraviolet radiation; ROS: Reactive Oxygen Species; GFs: Growth Factors; CKs: Cytokines; MEKK3,4: MAP3K3, MAP3K4; TAO: Serine/threonine-protein kinase Tao; ASK: Apoptosis signal-regulating kinase, MAP3K5; MLK1–3: Mitogen-Activated Protein Kinase Kinase Kinase 9–11; MKK4: Mitogen-Activated Protein Kinase Kinase 4; STAT1/3: Signal Transducer And Activator Of Transcription 1/3; ATF1,2: Activating Transcription Factor 1,2; cMYC: MYC Proto-Oncogene; ELK1: ETS Transcription Factor ELK1

Mitogen activated protein kinases (MAPKs) play vital roles in signalling transduction pathways and ability to control intracellular processes such as cell survival, differentiation, proliferation and apoptosis, via the sequential phosphorylation of substrate protein Ser/Thr kinase protein cascades. The three-tiered activation structure consists of the activation of a MAPK Kinase Kinase (MAP 3 K) followed by the phosphorylation of a MAPK Kinase leading to the dual phosphorylation of MAPK proteins [12]. The conventional subfamilies of MAPK include: the extracellular signal regulated kinase (ERK) proteins, ERK1/2 and ERK5 along with the stress activated Jun amino-terminal kinases (JNK) as well as the stress activated p38MAPK proteins (Fig. 1) [12, 13].

### p38β

The p38MAPK family is comprised of four homologous proteins: p38α, p38β, p38δ and p38γ and is involved in the integration of biochemical signals in response to environmental stresses as well as reactive oxygen species (ROS) and inflammatory cytokines [14, 15]. The p38MAPK family has been implicated in a number of cancer pathologies. p38α is the most widely studied of the isoforms and is ubiquitously expressed along with p38β, of which it shares around 75% homology, whereas p38δ and p38γ are differentially expressed throughout the tissues [16].

Over the last few decades, evidence implicating regulation of p38 with processes involved in cancer such as epithelial-mesenchymal transition, migration, invasion, and survival has grown. Until recently, investigation of p38 had primarily focused on the biomarker p38α, however mounting evidence also suggests roles for p38β and the other isoforms [17–20].

p38β is encoded by 12 exons of the gene MAPK11 that lie on chromosome 22 at the position 22:55,263,713-50,270,380 (UCSC genome browser/GRCh38/hg38). It is comprised of 364 amino acids and contains a kinase domain comprised of a T-G-Y dual phosphorylation motif that enables activity [21]. p38β is expressed in the vast majority the organs and cell types with high expression in endothelial cells yet low in those of hematopoietic origin such as macrophages and monocytes [22]. p38β is activated by MKK6 and expressed at a lower basal level while, p38α is activated by MKK3/6 often leading to the past characterisation of p38β as redundant [23]. p38α expression is crucial for foetal development and its absence is lethal, while expression of p38β is not [24–26]. Both p38α and p38β, along with the downstream MAPKs MK2/3/5, act as key regulators in gene expression as well as cell cycle progression and are activated in response to stress and many pathological processes including inflammation and cancer [27]. Unlike p38α, p38β modulates basal activity through self-activation via autophosphorylation [28]. While a lot of work has been done on the p38α expression and activity, the roles of p38MAPKs (β/γ/δ) remain to be elucidated.

Recent literature has presented emerging roles for p38β in tumour progression including the ability to regulate TGF-1β and VEGF growth factor mediated survival of endothelial cells in the absence of the proapoptotic p38α [29].

In addition, p38β is shown to play a significant role in the regulation of the oncogene lipocalin 2 (LCN2) a target of plakophilin 3 (PKP3). Where PKP3 expression is decreased p38β is capable of regulating LCN2 leading to an increase in tumour invasion and metastasis [30]. In all cancers, an increasing number of key proteins or long non-coding RNAs are also starting to be associated with p38β expression and protein-protein interaction [31–35]. The Long non-coding RNA, LINCO1220 in particular, has received interest due to its roles in p38β suppression and in endometrial cancer and as such its therapeutic potential [33].

Despite the numerous studies investigating the expression and role of p38β in cancers such as lung, Head and Neck Squamous Cell carcinoma as well as prostate the role in female tissue specific cancers remains elusive [36, 37]. As such, this bioinformatic review will provide an overview of the role of p38β in all cancers with a detailed focus of p38β in female tissue specific cancers.

### p38β in female cancers

#### Breast cancer (BRCA)

p38MAPKs translate extracellular signals to intracellular response. Their role in cell proliferation, differentiation and response to inflammation is vital for normal cell function. They are regulators of major pathways including the ERK1/2-JNK (extracellular signal regulated-kinase, c-Jun N-Terminal Kinase) pathways which are.

both associated with poor clinical outcome in breast cancer patients [38]. Despite the viability of a p38β^−/−^ knock out mice and the consideration of p38α as the predominant isoform in response to cellular stresses there is increasing evidence of p38β involvement in stress response and cancer cell biology [39, 40]. Upregulation of p38β in breast cancer is a prime example, p38β regulation may not have been linked to tumour growth however increased expression is associated with upregulation of monocyte chemotactic protein-1 (MCP-1) leading to osteoclast differentiation and promotion of bone metastasis in breast cancer patients [40]. Apart from the following gynaecological cancers, although rare (~ 1%), breast cancer may affect men as well. Male breast cancer is often associated with inherited mutations of BRCA1, BRCA2 and PIK3CA genes, obesity, estrogen treatments (hormonal therapies for prostate cancer) and Klinefelter syndrome [41–43].

#### Ovarian cancer (OV)

It has been found that Galectin-1 (Gal-1) is an indicator of poor prognosis in ovarian cancer patients where high expression of Gal-1 is observed in ovarian cancers of higher histological grade as well as advanced lymph node status [44, 45]. Activation of the MAPK JNK/p38 pathway is influenced by Gal-1 and facilitates epithelial-mesenchymal transition (EMT) making it a promising target for prevention of epithelial ovarian cancer metastasis [45]. In addition to Gal-1, overexpression of genes such as LAMC2 are also associated with increased cell proliferation, repression of cell apoptosis and increased expression of p38. Inhibition of p38 nuclear accumulation through the negative regulation of LAMC2 by miR-125a-5p is also shown to repress tumorigenesis in epithelial ovarian cancer [46].

#### Uterine Corpus endometrial carcinoma (UCEC)

Uterine Corpus Endometrial Carcinoma has not been investigated in as much detail, but a few markers have been identified that play significant roles in the progression of this type of cancer. Mutation of the PTEN gene, for example, affects cellular signalling through inhibition of MAPK pathway and is associated with histological subtype as well as the early stage characteristics of endometrial cancer. Regulation of cell proliferation - differentiation by MAPK via RAF-MEK-ERK may provide a targetable mechanism for future research [47]. Studies have shown that oestradiol is a potential activator of the MAPK pathway through estrogen (ER) and Insulin receptor (InsR) interaction. Insulin synergistically with ER activates phosphorylation of MAPK and PI3K pathways [48, 49]. Phosphorylated Akt (p-Akt), mTOR (p-mTOR) and MAPK (p-MAPK) proteins as well as lncRNAs like HEIH, are often implicated in endometrial carcinogenesis and tolerance of common antineoplastic agents such as paclitaxel [50, 51]. As such, MAPK and PI3K/Akt are promising targets of new anti-tumor agents such as emodin (rhubarb) [47].

#### Uterine Carcinosarcoma (UCS)

Uterine carcinosarcoma is usually classified as uterine endometrial carcinoma but is often more aggressive than UCEC. Little is known about the molecular effects that influence aggressive biphasic growth of the sarcomatous elements that account 2–5% of the uterine corpus malignancies [52, 53]. It has been shown that UCSs undergo EMT and highly express signal transducers such as TGF-β; SMAD2/3, playing an important role in the regulation of cell growth and development [54]. There is very little evidence regarding investigation of MAPK and other signalling pathways in UCS within the literature, in light of the involvement of MAPK signalling in other female malignancies this absence presents an area for further investigation.

#### Cervical squamous cell carcinoma (CESC)

In Cervical Squamous Cell Carcinoma, activation of p38 when driven by mediators such as osteopontin (OPN) leads to invasive progression. Phosphorylated p38 by CD44-mediated MKK3/6, high expression of OPN and furin, induction of NF-κB and p65 are correlated with cervical cancer progression and are considered therapeutic targets [55]. Not only is p38 inhibition a target of novel anti-tumour agents, there are also reports of suppression through use of traditional herbal medicines such as Matrine, which consequently also decreases the expression of matrix metalloproteinases, MMP-2 and MMP-9 [56]. In contrast, activation of MMPs via p38/NFκB pathway with inflammatory cytokines like interleukin 17A (IL-17A), is associated with the invasion of cervical cancer cells, making IL-17A another potential prognosis marker of CESC [57]. Despite the tumorigenic nature of p38 there is evidence of dual role status as both a tumour suppressor and protooncogene; Xanthonoids such as Alpha-mangostin for example elevate reactive oxygen species (ROS) as well as p38 and consequently damage mitochondrial integrity while instigating apoptosis within cervical cancer cells [58].

Using large-scale data sets such as TCGA, genetic analysis and potential biomarker identification is becoming increasingly accessible to researchers. *In-silico* analysis using online datasets and tools can further the understanding of alterations and mechanisms which influence expression such as the mutational status and methylation profile of genes of interest without the need of expensive and time consuming in-vivo*/*in-vitro analysis. This research seeks to use data visualization tools compiling data from TCGA for the analysis of the p38MAPK isoform p38β in cancers of female reproductive tissue origin, presenting evidence for the further exploration of this signalling molecule in cancer cell biology for molecular understanding and potential therapeutic translation.

## Results

**Fig. 2:**
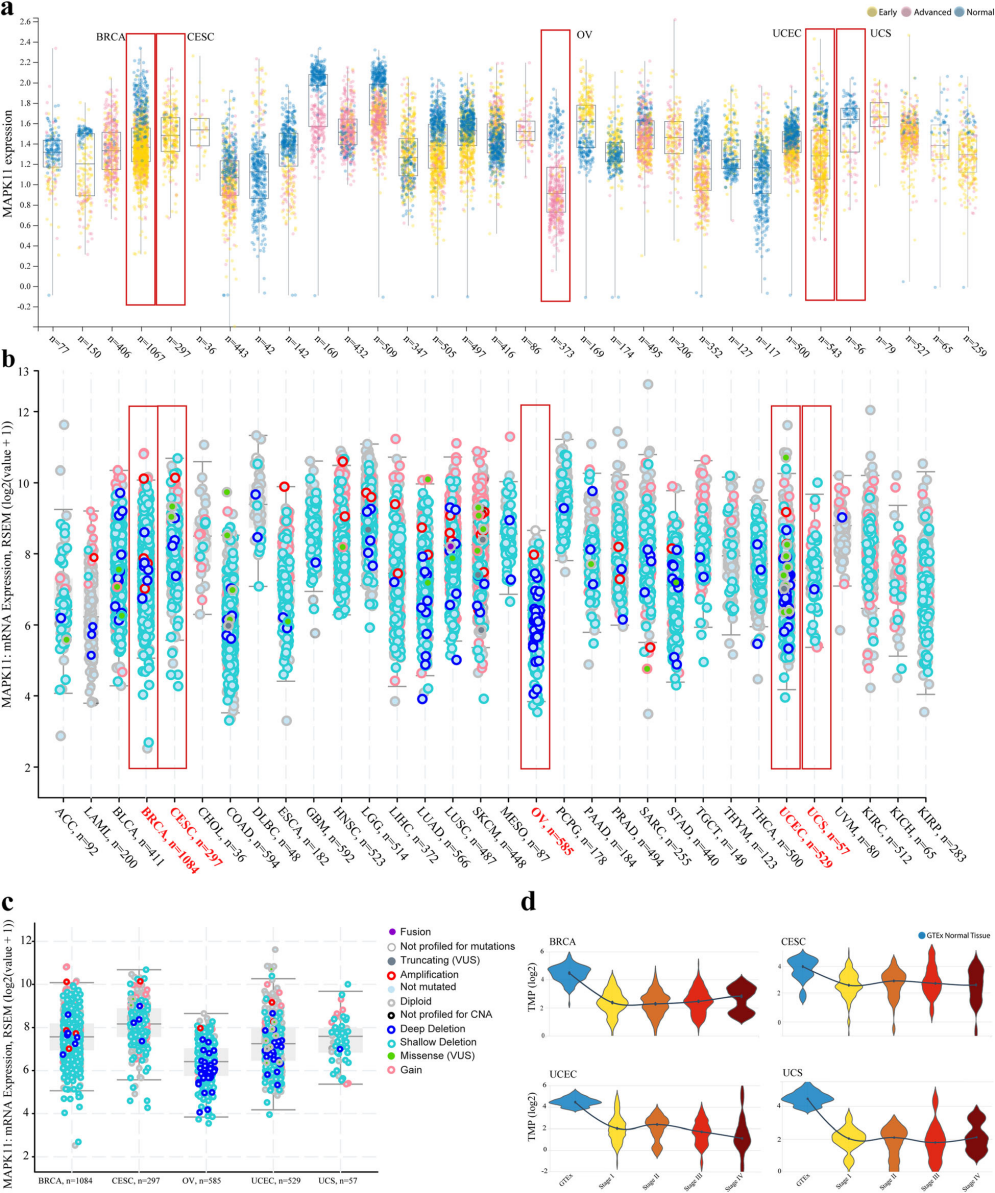
**a** PanCancer expression analyses of MAPK11 from canSAR database. A higher expression is seen in normal (blue) samples from female data sets: BRCA, OV, UCEC, and UCS compared with early (yellow) and advanced stage (pink) samples, with OV exhibiting the lowest level of expression in the advanced stage compared to all cancer data sets; **b** PanCancer data analysis using cBioPortal provides an overview of the landscape of MAPK11 genetic alterations across the range of TCGA data sets. OV, UCEC exhibit the highest level of alteration within the cancer types with deep deletion presenting as the most prevalent throughout the data sets. Of the female-specific cancers: BRCA, OV, CESC, UCEC, each exhibit varying levels of amplification specific alterations as well as high levels of deep deletion; **c** Deep deletions in BRCA 7/1084, CESC: 4/297, OV: 28/585, UCEC: 22/529 and UCS: 1/57. OV and the UCEC patients with deep deletions of MAPK11 are the biggest populations compared to all other cancers; **d** MAPK11 expression in female tissues: BRCA, CESC, UCEC, and UCS, indicating higher expression in normal tissue compared to downregulation in cancer data sets across all four cancer stages

The PanCancer analysis of MAPK11 provides an overview of expression throughout the early and advanced stages of the disease as well as the genetic alterations of MAPK11 that contribute towards changes in expression. Focusing on female tissue-specific cancers (Fig. 2a), we outline a trend where MAPK11 expression in normal tissue is higher than that of early-stage BRCA, CESC, UCEC, and UCS cancer data sets. Unlike the other female cancers, MAPK11 expression in OV is primarily shown at the advanced stage of the disease which is often the stage of diagnosis due to the ambiguity of symptom presentation (Fig. 2d). The average expression of MAPK11 in OV (0.9 RSEM (log2(value + 1)), is relatively low compared to the other female cancers and is mirrored in the low expression alteration profile seen in Fig. 2b where shallow and deep deletion are the most frequent type of alteration.

### Gene expression and methylation

It is generally considered that DNA methylation within the CpG island region of a gene’s promoter region is correlated with gene expression while DNA methylation within the gene body is also associated with chromosomal integrity [59]. To analyse the methylation status of MAPK11, 14 probes were chosen through the SMART database and studied within the female cancers: BRCA, CESC, and UCEC (Table 1).

**Table 1:** List of the 14 probes analysed

Probe	Chromosome	Start	End	CGIposition	CpG
cg26790091	chr22	50,264,031	50,264,032	S_Shelf	
cg03717414	chr22	50,265,420	50,265,421	N_Shore	
**cg23755154**	chr22	50,266,978	50,266,979	Island	**CpG1**
**cg15036874**	chr22	50,267,519	50,267,520	Island	
**cg16054907**	chr22	50,267,636	50,267,637	Island	
**cg13577505**	chr22	50,267,819	50,267,820	Island	
cg06018119	chr22	50,268,221	50,268,222	S_Shore	
**cg19184963**	chr22	50,270,210	50,270,211	Island	**CpG2**
**cg08211722**	chr22	50,270,495	50,270,496	Island	
**cg15554007**	chr22	50,270,507	50,270,508	Island	
**cg00735239**	chr22	50,270,518	50,270,519	Island	
**cg00164898**	chr22	50,270,698	50,270,699	Island	
**cg00395632**	chr22	50,270,792	50,270,793	Island	
cg11070772	chr22	50,272,060	50,272,061	S_Shore	

**Fig. 3:**
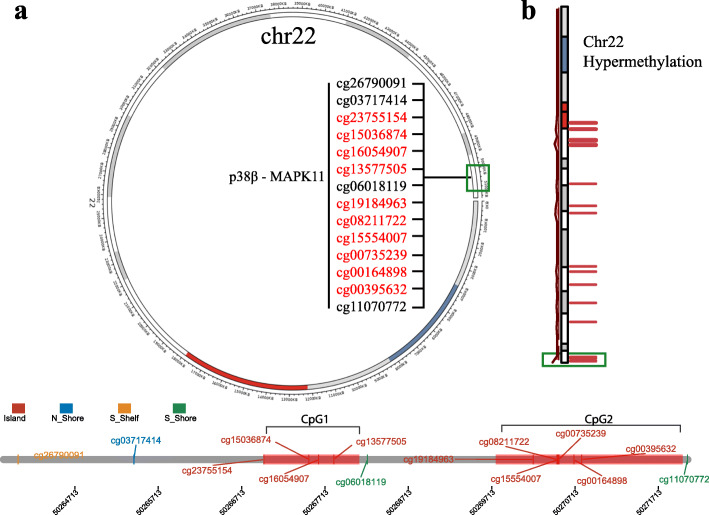
**a** Genomic information of the gene MAPK11. The segment plot showing the detailed information of genomic locations of CpGs of MAPK11, highlighting CpG island, shelves, and shores. The name and the type of each transcript are given. The coverage of the CpG islands is displayed as the red region. 14 probes are included in this genomic location with 10 of them to lie on the two island regions (CpG1 and CpG2); **b** Red bars on chromosome 22 indicate hypermethylated regions. MAPK11 gene can be found in the right end of the chromosome at the cytogenetic band 22q13.33 (green box)

Here, we find a positive correlation of MAPK11 expression with methylation of the promoter region. Two of these CpG islands were found within the genomic region of MAPK11. The probe cg26790091 at the S-Shelf region showed lower methylation values compared to normal datasets and a positive correlation of expression and methylation was seen for all datasets with the exception of UCS (R = -0.04). The same pattern is observed with the N-Shore probe cg03717414, with lower methylation in all tested cancers compared to normal and a positive correlation between expression and methylation, again with exception to UCS (R = -0.0053). Following the genomic region of MAPK11, we found 10 locations on 2 islands with the first island having 4 methylation targets (CpG1: cg23755154, cg15036874, cg16054907, cg13577505) to present a strong positive correlation of expression-methylation for all 4 cancers (exception is probe cg15036874 in BRCA with an R = -0.095). Following the S-shore region, the next 6 locations located within the second island exhibit hypomethylation with a strong correlation between expression and methylation status (CpG2: cg19184963, cg08211722, cg15554007, cg00735239, cg00164898, cg00395632). Detailed results are located in Additional files 1, 2, and 3. Differences between the observed levels of expression and region-specific methylation are seen in Fig. 3 may influence the binding of transcriptional factors at the promoter region of the gene as well as the stability of the gene itself [60].

Gene expression and methylation analysis show that in BRCA, methylation at cg23755154 is significantly increased while the hypomethylated cg19184963 and cg00395632 are decreased within the tumour dataset (Additional file 1). In CESC, the dataset exhibits significant hypermethylation at cg15036874 and cg16054907 along with hypomethylation of the aggregated probes with tumour showing higher levels of methylation compared to normal tissue (Additional file 2). In UCEC, significant probe sites include the hypermethylation of cg23755154 and cg16054907 (Additional file 3). Information about the UCS and OV aggregation could not be obtained due to limitations of the SMART analysis tools (see Additional file 4).

**Fig. 4:**
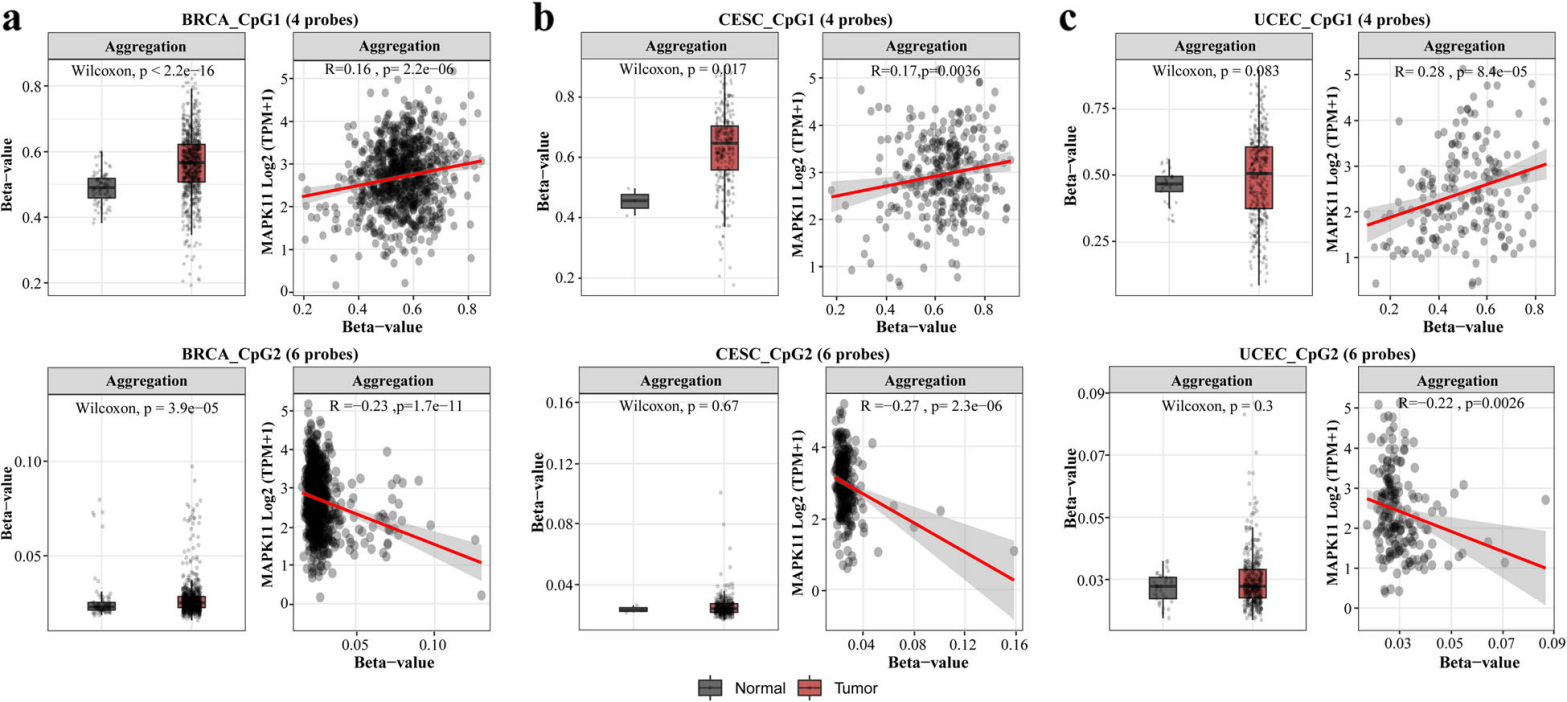
MAPK11 Methylation and correlation of expression with methylation in **a** BRCA; **b** CESC; **c** UCEC. In the top, the CpG1 island with 4 probes is showing a clear positive correlation of the expression of MAPK11 with the methylation status, while at the bottom, the CpG2 island with 6 probes, is showing a clear negative correlation of the expression with the methylation. Overall, a high methylation status has been observed in the first 4 positions targeted by the probes and lower methylation was observed in the following island, indicating that the gene is split into 2 genomic regions of different methylation status (see more at Additional files 1, 2 and 3)

We chose to group the methylated probes by region separating those that are located at CpG island 1 and those at CpG island 2 to analyse the separate promoter binding regions. A Spearman correlation analysis between the expression of MAPK11 and DNA methylation (M value) of MAPK11 in three of the female cancers indicates that expression is significantly positively associated with the methylation of region 1, a phenomenon that does not align with the normal paradigm of gene expression (cg23755154, cg15036874, cg16054907,cg13577505). Negative correlation however was seen with the following 6 probes that are localised to the second CpG island: cg19184963, cg08211722, cg15554007, cg00735239, cg00164898, cg00395632 (Fig. 4, see Additional files 1, 2, and 3).

Average aggregation of probes indicates that the gene is hypermethylated within the region of the first CpG island region yet hypomethylated in the second for both cancer data and normal, there is little significant difference between the levels of expression and methylation for the aggregated probes of BRCA with CESC and UCEC only exhibiting slight variation. Further analysis of the collection of probes aligning at the CpG island regions of the gene MAPK11 in wildtype and cancer data sets can be seen in Additional Fig. 5.

**Fig. 5:**
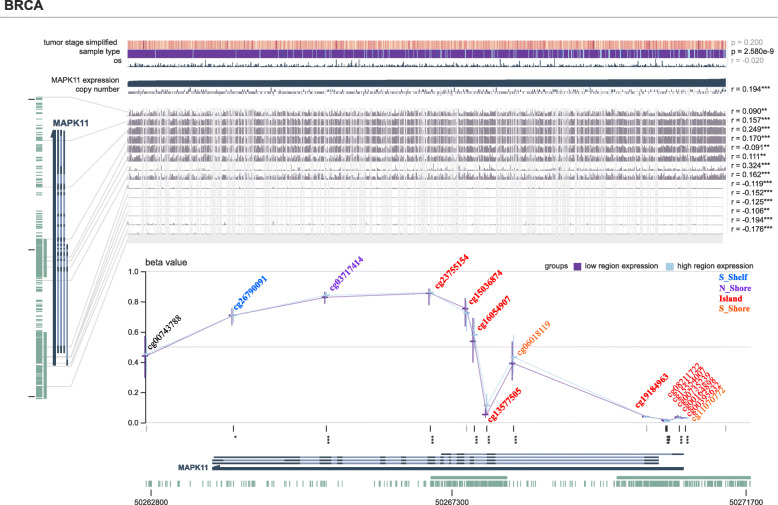
MEXPRESS view of the TCGA data for MAPK11 in breast invasive carcinoma. The samples are ordered by MAPK11 expression, revealing that the expression in the different stages is not significantly altered. Probes have been coloured differently depending on the genomic region they target. The detailed analysis for all cancers analysed can be found in Additional file 5

Complementary methylation analysis using MEXPRESS, indicates that there is no significant difference in the expression of the MAPK11 between the stages of each cancer data set studied (Fig. 5, Additional file 5). Moreover, no significant changes were observed for the BRCA oestrogen and BRCA progesterone receptor status, HER2/neu receptor, or menopause status of the cancers (data not shown here).

## Methods

**MEXPRESS** web tool was used to integrate TCGA data for the visualisation of gene specific methylation and expression of MAPK11 in relation to genomic location and clinical data such as age, weight and receptor status [61].

DNA methylation is a reversible process consisting of the covalent binding of a methyl group to cytosine, one of four DNA bases. In humans, DNA methylation is almost exclusively restricted to the cytosines of CpG dinucleotides and plays a critical role in the regulation of gene expression. Abnormal DNA methylation patterns are found ubiquitously in all types of human cancer. The precise genomic location of DNA methylation is one of the most important regulatory factors of gene expression, and is where MEXPRESS distinguishes itself from other available tools [62].

**Shiny Methylation Analysis Resource Tool (SMART)** provided access to a comprehensive overview of MAPK11 DNA methylation and associated omics data, through integration of TCGA data [63, 64]. This application was used to correlate epigenetic modifications with gene expression in cancer data sets using probes specific to genomic regions of MAPK11. The following 14 probes were used in analysis of MAPK11 (Τable 1).

The **cBioPortal** (www.cbioportal.org/) which contained both sequencing and pathological data on 30 different cancers was used to analyze the genetic alteration of MAPK11 across different cancer types [65, 66]. The Breast Invasive Carcinoma (TCGA, PanCancer Atlas, *n* = 1075), Cervical Squamous Cell Carcinoma (TCGA, PanCancer Atlas, *n* = 297), Ovarian Serous Cystadenocarcinoma (TCGA, PanCancer Atlas, *n* = 585), Uterine Corpus Endometrial Carcinoma (TCGA, PanCancer Atlas, *n* = 529), Uterine Carcinosarcoma (TCGA, PanCancer Atlas, *n* = 57) datasets were selected for further analyses of MAPK11 expression and mutation status [65, 66].

**canSAR**. Pan-cancer analysis of the MAPK11 expression and expression in the different cancer stages of the four female cancers was conducted through canSAR (cansar.icr.ac.uk), a public, freely available, integrative translational research and drug discovery knowledge base [67].

## Discussion

The MAPK signalling cascades are integral signalling mechanisms that play an active role in cellular processes including proliferation, apoptosis, differentiation, and cell development. The p38MAPK isoforms, in particular, mediate response to external stresses, inflammatory cytokines as well as reactive oxygen species (ROS) [68–70].

Until recently, clinical evaluation of a family of key regulators, the p38MAPK isoforms have widely focused on the study and consideration of p38α as a biomarker of cancer [71]. However, recent evaluations of p38 implicate the additional isoforms with cancer cell biology. The expression of p38β has received growing interest for its roles in female cancers such as breast and endometrial [33].

Data generated within this study sought to explore the expression of p38β in the female tissue-specific cancers: BRCA, CESC, OV, UCEC, and UCS, revealing down-regulated expression of the gene in each cancer data set compared to normal tissue (Fig. 2). Deep deletions within the MAPK11 gene were the most frequent type of alteration within the gene and no significant difference of expression seen between stages of the cancers studied (TCGA data regarding OV is not as extensive as the other data sets). These trends in the expression are converse to that of p38α which is often over-expressed within cancer cells and considered to render the roles of p38β redundant. However, recent publications suggest that p38β is capable of self-activation through autophosphorylation and the regulation of specific pathways outlined in the introduction [28, 72].

Regulation of genes is often influenced through methylation. Using the SMART analysis tool, the methylation status of MAPK11 at specific regions was assessed. Grouped analysis of the methylation probes specific to the two CpG islands located on the MAPK11 gene was used to reveal the methylation status and expression of the gene promoter regions. Hypermethylation of the gene in the CpG1 was seen for BRCA, CESC, and UCEC with an increase in the methylation of the gene in tumour samples as well as a positive correlation with expression. This trend does not follow the general paradigm often seen with hypermethylation and gene suppression suggesting that methylation is not the only regulator of gene expression and that there are additional transcriptional and post-transcriptional factors influencing the decreased levels of p38β expression within these cancers. The CpG2 island however displays hypomethylation of the region along with negative correlation and relatively high levels of gene expression with little difference between cancer and normal.

The differential methylation of specific regions of a gene is often associated with both expression and gene stability. Therefore, the different levels of methylation within the CpG islands of MAPK11 between normal and cancer data sets may influence the decrease in expression of MAPK11 seen in cancer tissues and warrants further investigation as a possible biomarker (Figs. 2, 3, 4) [60].

Additional analysis through MEXPRESS was also used to assess the correlation between the receptor status and expression of MAPK11. The oestrogen receptor alpha is thought to mediate the development of endometrial cancer and is a biomarker of breast cancer. However, no differences in expression were seen relative to oestrogen, progesterone, or HER2/neu receptor status as well as menopausal status suggesting that p38β activation is not mediated via these pathways that are often associated with female cancers (Additional file 5) [48].

Despite the decreased level of expression, the high level of deep deletion within the gene and differences in methylation may influence post-transcriptional regulation and phosphorylation of the gene, influencing its ability to self-activate. For example, therapeutics such as emodin which target phosphorylation are shown to maintain basal levels of genes involved in the p38MAPK pathway while inducting ROS activated apoptosis of cancer cells [47, 73].

Activation of p38β via the transcription factor Pokemon/Zbtb7 is associated with tumorigenesis and cell invasion in HepG2 cells [30]. Downstream signalling of p38β also leads to an increase in expression of the oncogene, Lipocalin2 (LCN2), and an increase in tumour cell migration [17, 30]. Additional downstream targets include mediators of proliferation and survival such as AP-1 and mTOR which consequently are also involved in gynaecological malignancy [74, 75]. MAPK11 has shown oncogenic properties through increased regulation of the WNT inhibitor, Dickkopf WNT signalling pathway inhibitor 1 (DKK-1), a key regulator of bone metastasis [31]. Elevated levels of DKK-1 are also associated with advanced clinical stage and poor prognostic outcomes in gynaecological malignancies including ovarian cancer [76]. The upregulation of p38β through lncRNA 1220 is also associated with endometrial cancer through the mediation of proliferation and inhibition of apoptosis. p38β phosphorylates Myocyte Enhancer Factors (MEF) such as MEF2A and MEF2C regulators of differentiation, proliferation, apoptosis, migration as well as metabolism especially in the absence of p38α [77]. It has been shown that it increases osteoclast differentiation and activity through upregulation of monocyte chemotactic protein-1 (MCP-1) [40, 78]. It has also been suggested that the p38MAPKS are capable of acting as both a tumour suppressor as well as an oncogene [79–81]. With the lower expression levels, its ability to regulate these vital cell functions and act as a tumour suppressor may be compromised.

Further investigation of the role in these female tissues may provide an opportunity for uncovering a duel role of MAPKp38 isoforms as both oncogenes and tumour suppressors in cancer cell biology.

## Supplementary Information

**Additional file 1:.** MAPK11 Methylation and correlation of expression with methylation in BRCA. In the top, the CpG1 island with 4 probes is showing a clear positive correlation of the expression of MAPK11 with the methylation status, while at the bottom, the CpG2 island with 6 probes, is showing a clear negative correlation of the expression with the methylation. Overall, a high methylation status has been observed in the first 4 positions targeted by the probes and lower methylation was observed in the following island, indicating that the gene is split into 2 genomic regions of different methylation status.

**Additional file 2:.** MAPK11 Methylation and correlation of expression with methylation in CESC. In the top, the CpG1 island with 4 probes is showing a clear positive correlation of the expression of MAPK11 with the methylation status, while at the bottom, the CpG2 island with 6 probes, is showing a clear negative correlation of the expression with the methylation. Overall, a high methylation status has been observed in the first 4 positions targeted by the probes and lower methylation was observed in the following island, indicating that the gene is split into 2 genomic regions of different methylation status.

**Additional file 3:.** MAPK11 Methylation and correlation of expression with methylation in UCEC. In the top, the CpG1 island with 4 probes is showing a clear positive correlation of the expression of MAPK11 with the methylation status, while at the bottom, the CpG2 island with 6 probes, is showing a clear negative correlation of the expression with the methylation. Overall, a high methylation status has been observed in the first 4 positions targeted by the probes and lower methylation was observed in the following island, indicating that the gene is split into 2 genomic regions of different methylation status.

**Additional file 4:.** MAPK11 Methylation and correlation of expression with methylation in BRCA, CESC, UCEC and UCS of all the 14 probes.

**Additional file 5:.** Detailed presentation of methylation status of the MAPK11 gene in BRCA, CESC, UCEC and UCS as retrieved from MEEXPRESS analysis. The CpG island probes are presented in red and asterisks annotations represent statistical significance.

**Additional file 6:.** Heatmap of all 14 methylated MAPK11 probes in BRCA, CESC, UCEC and UCS. The CpG island probes are presented in red.
