# Supplementary figures and images for: p38β - MAPK11 and its role in female cancers
Source: J Ovarian Res. 2021 Jun 26;14:84. doi: 10.1186/s13048-021-00834-9 (PMC8236201; doi:10.1186/s13048-021-00834-9)

## BRCA\_CpG1

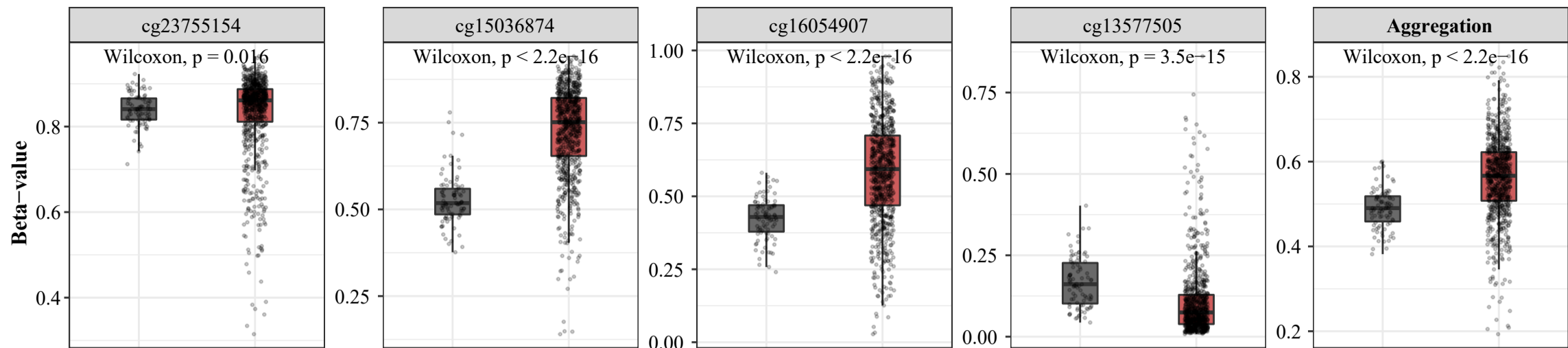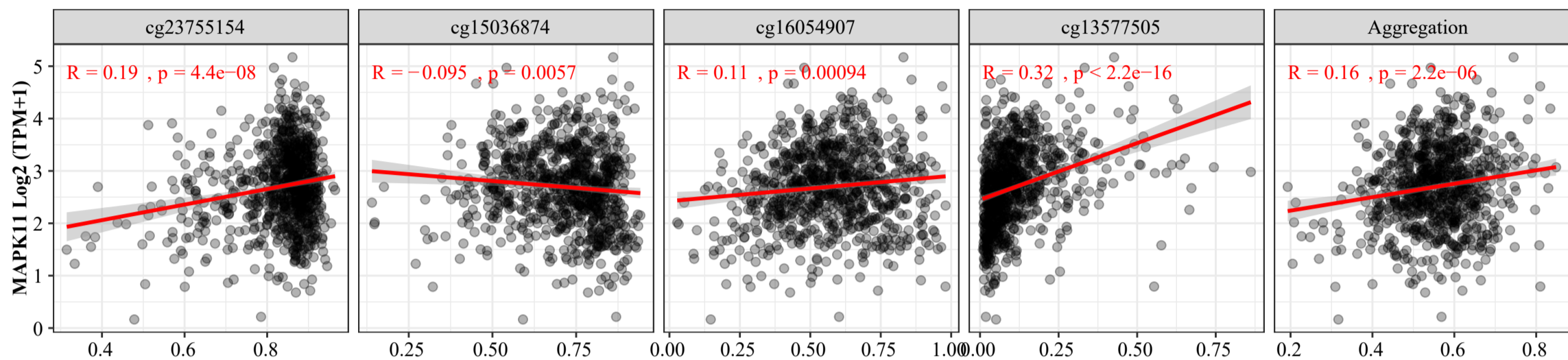

## BRCA\_CpG2

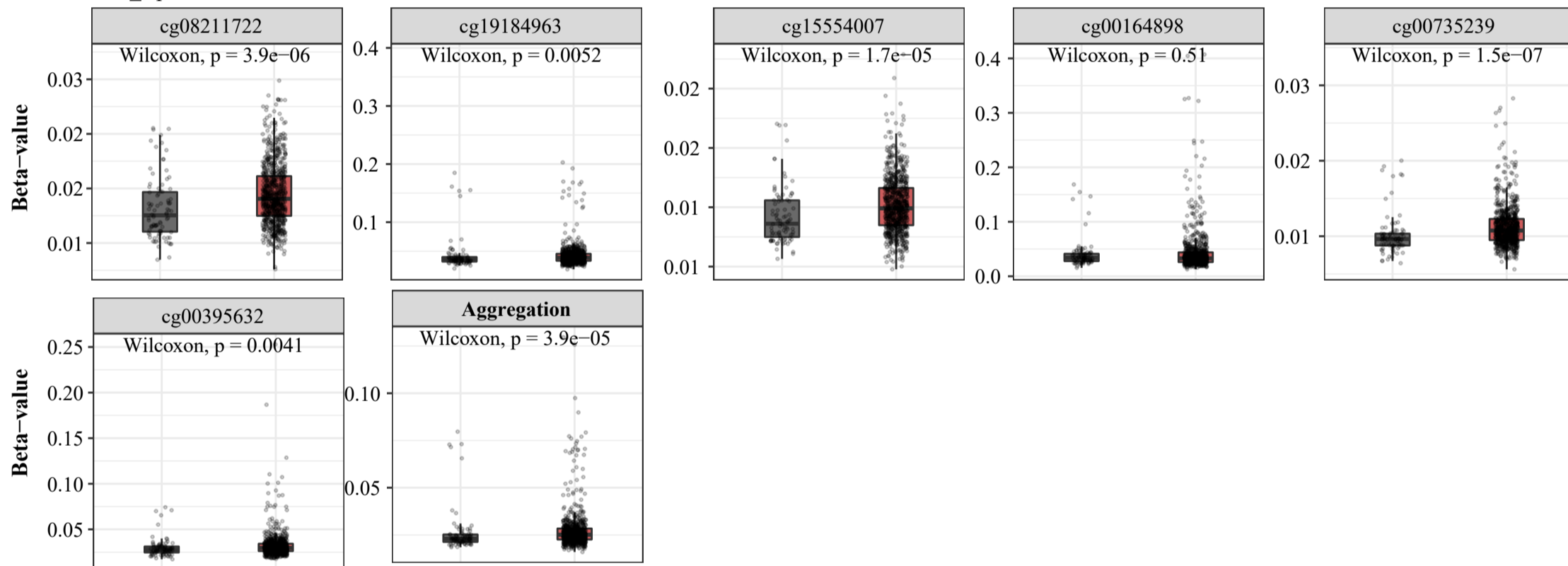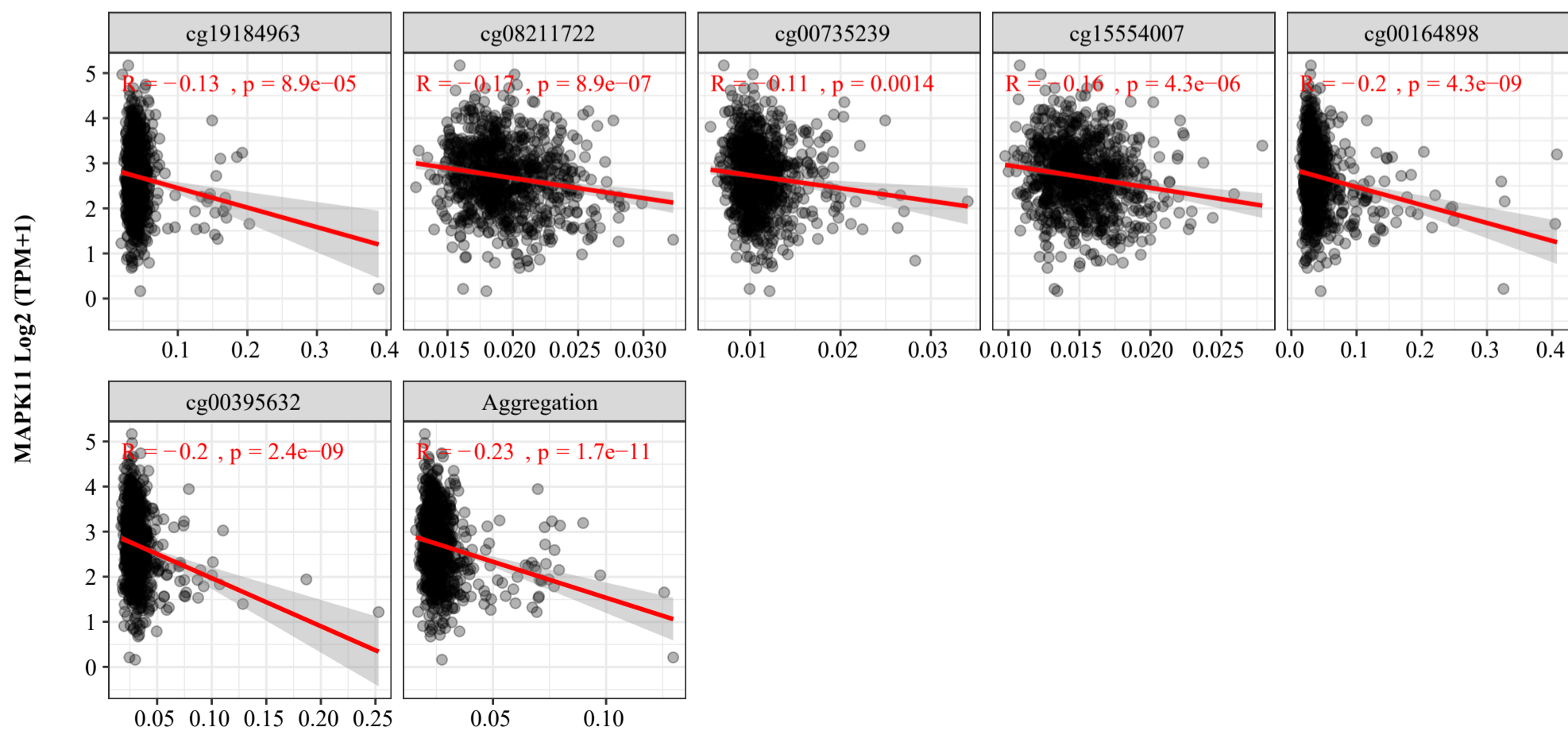

Supplement: Supplementary file 1 — Additional file 1:. MAPK11 Methylation and correlation of expression with methylation in BRCA. In the top, the CpG1 island with 4 probes is showing a clear positive correlation of the expression of MAPK11 with the methylation status, while at the bottom, the CpG2 island with 6 probes, is showing a clear negative correlation of the expression with the methylation. Overall, a high methylation status has been observed in the first 4 positions targeted by the probes and lower methylation was observed in the following island, indicating that the gene is split into 2 genomic regions of different methylation status. [file 13048_2021_834_MOESM1_ESM.pdf]

## CESC\_CpG1

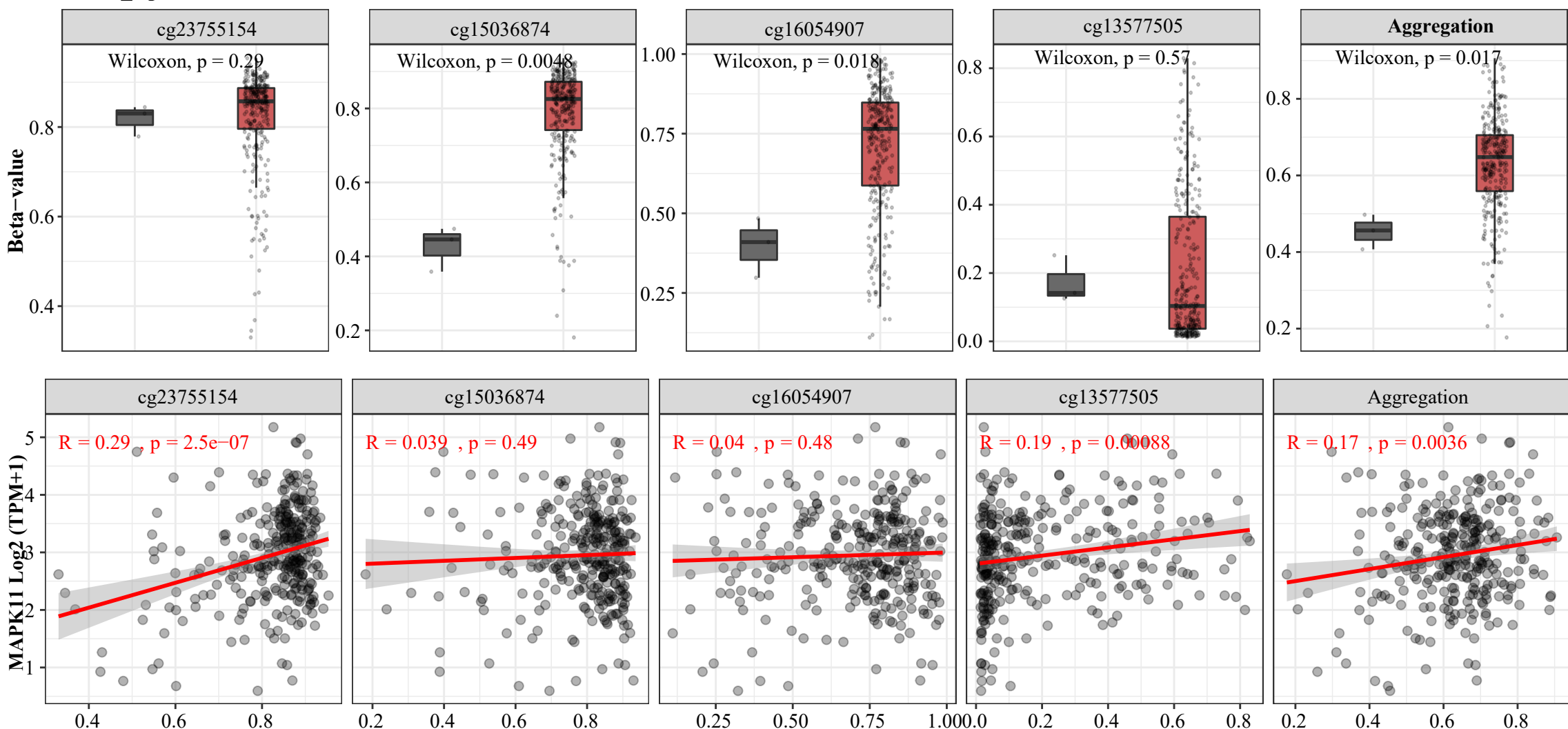

## CESC\_CpG2

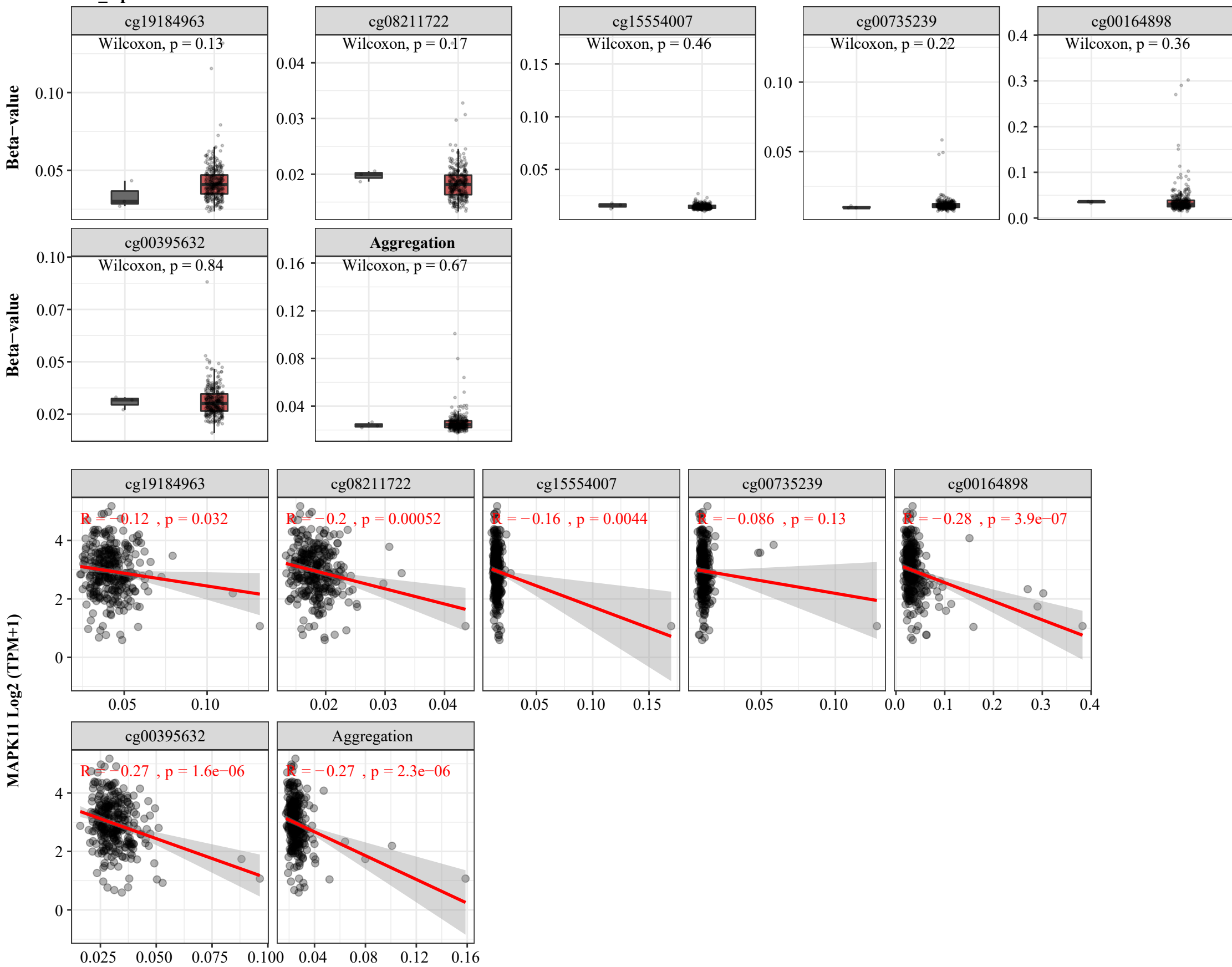

Supplement: Supplementary file 2 — Additional file 2:. MAPK11 Methylation and correlation of expression with methylation in CESC. In the top, the CpG1 island with 4 probes is showing a clear positive correlation of the expression of MAPK11 with the methylation status, while at the bottom, the CpG2 island with 6 probes, is showing a clear negative correlation of the expression with the methylation. Overall, a high methylation status has been observed in the first 4 positions targeted by the probes and lower methylation was observed in the following island, indicating that the gene is split into 2 genomic regions of different methylation status. [file 13048_2021_834_MOESM2_ESM.pdf]

### UCEC\_CpG1

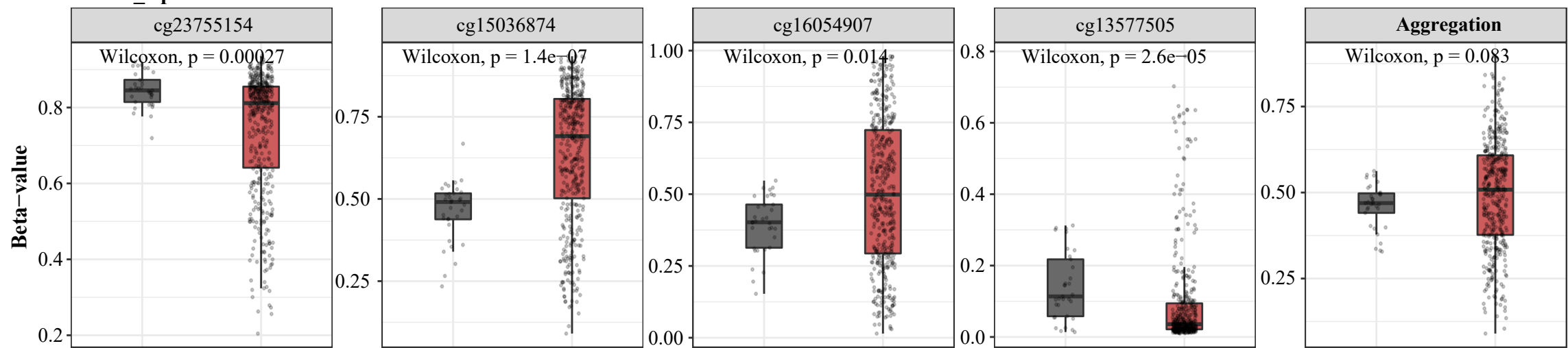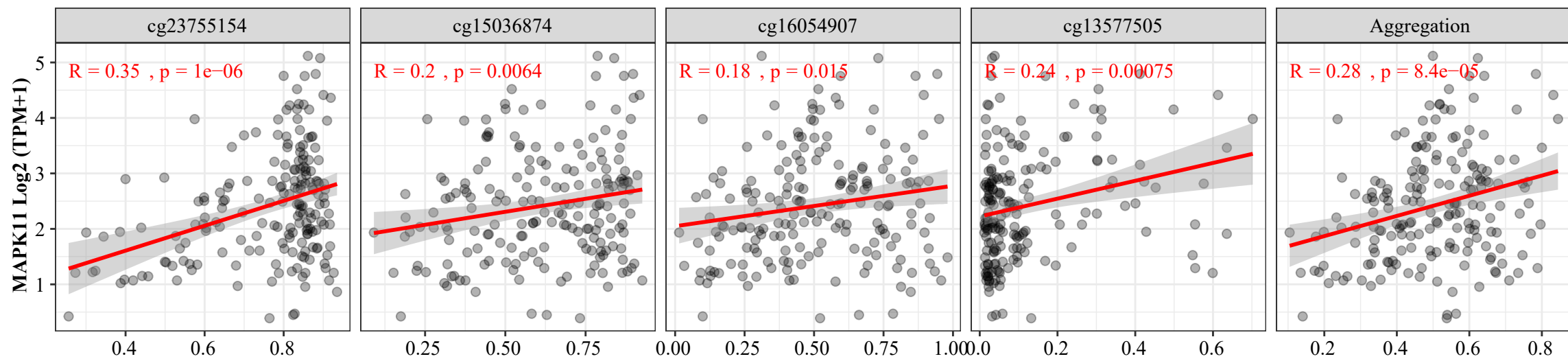

### UCEC\_CpG2

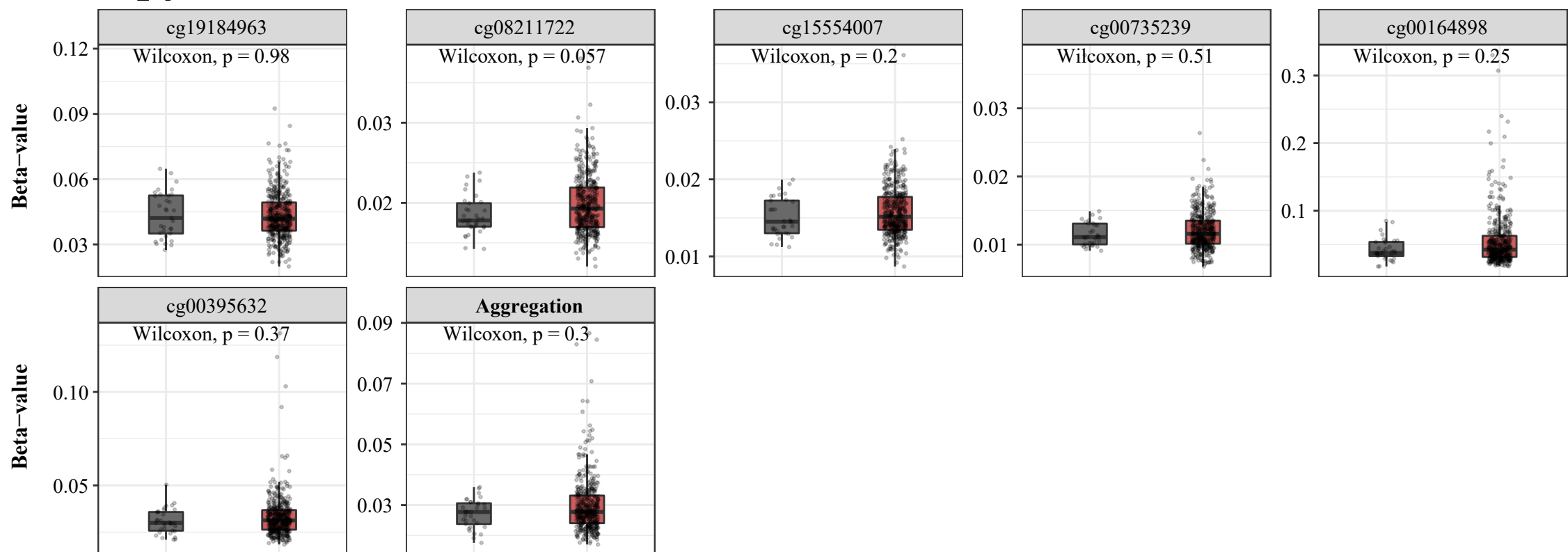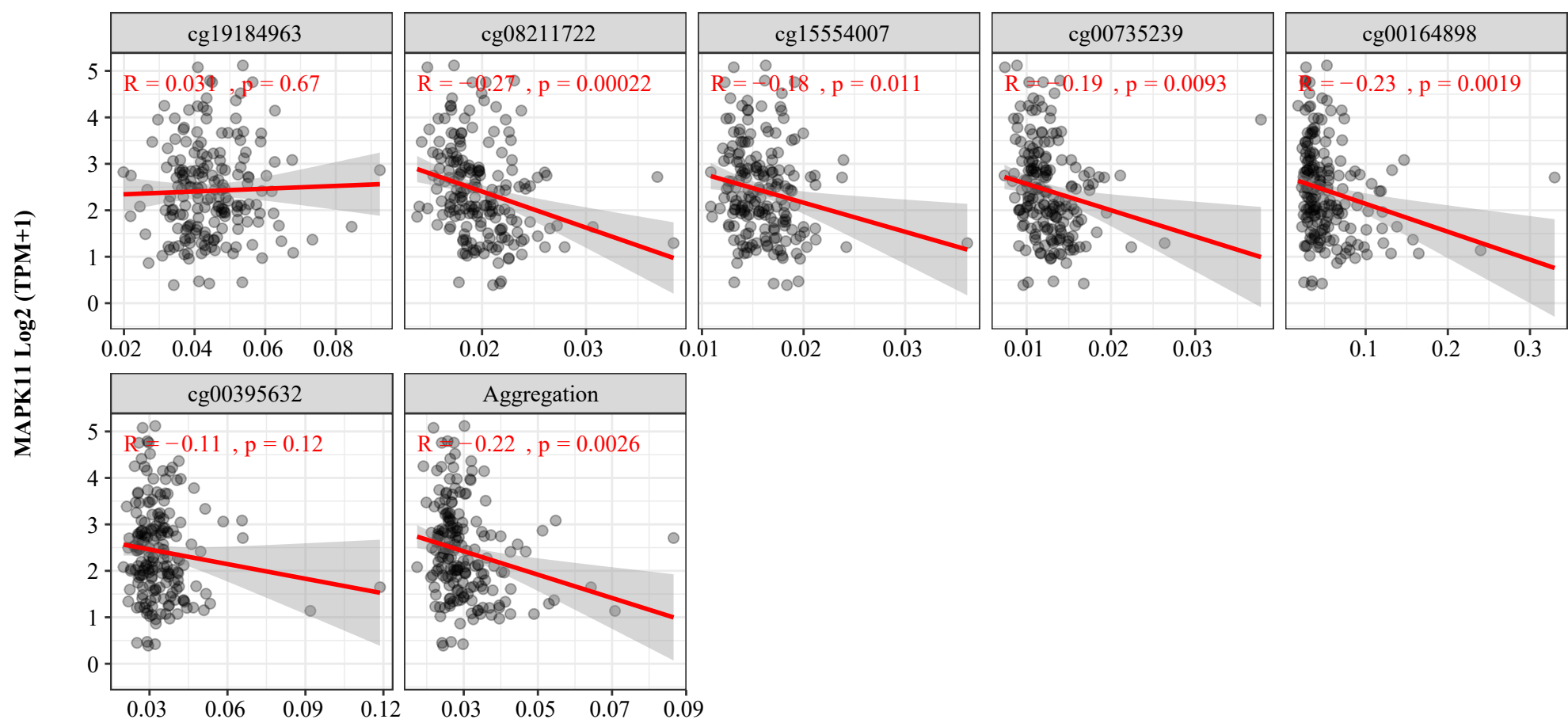

Supplement: Supplementary file 3 — Additional file 3:. MAPK11 Methylation and correlation of expression with methylation in UCEC. In the top, the CpG1 island with 4 probes is showing a clear positive correlation of the expression of MAPK11 with the methylation status, while at the bottom, the CpG2 island with 6 probes, is showing a clear negative correlation of the expression with the methylation. Overall, a high methylation status has been observed in the first 4 positions targeted by the probes and lower methylation was observed in the following island, indicating that the gene is split into 2 genomic regions of different methylation status. [file 13048_2021_834_MOESM3_ESM.pdf]

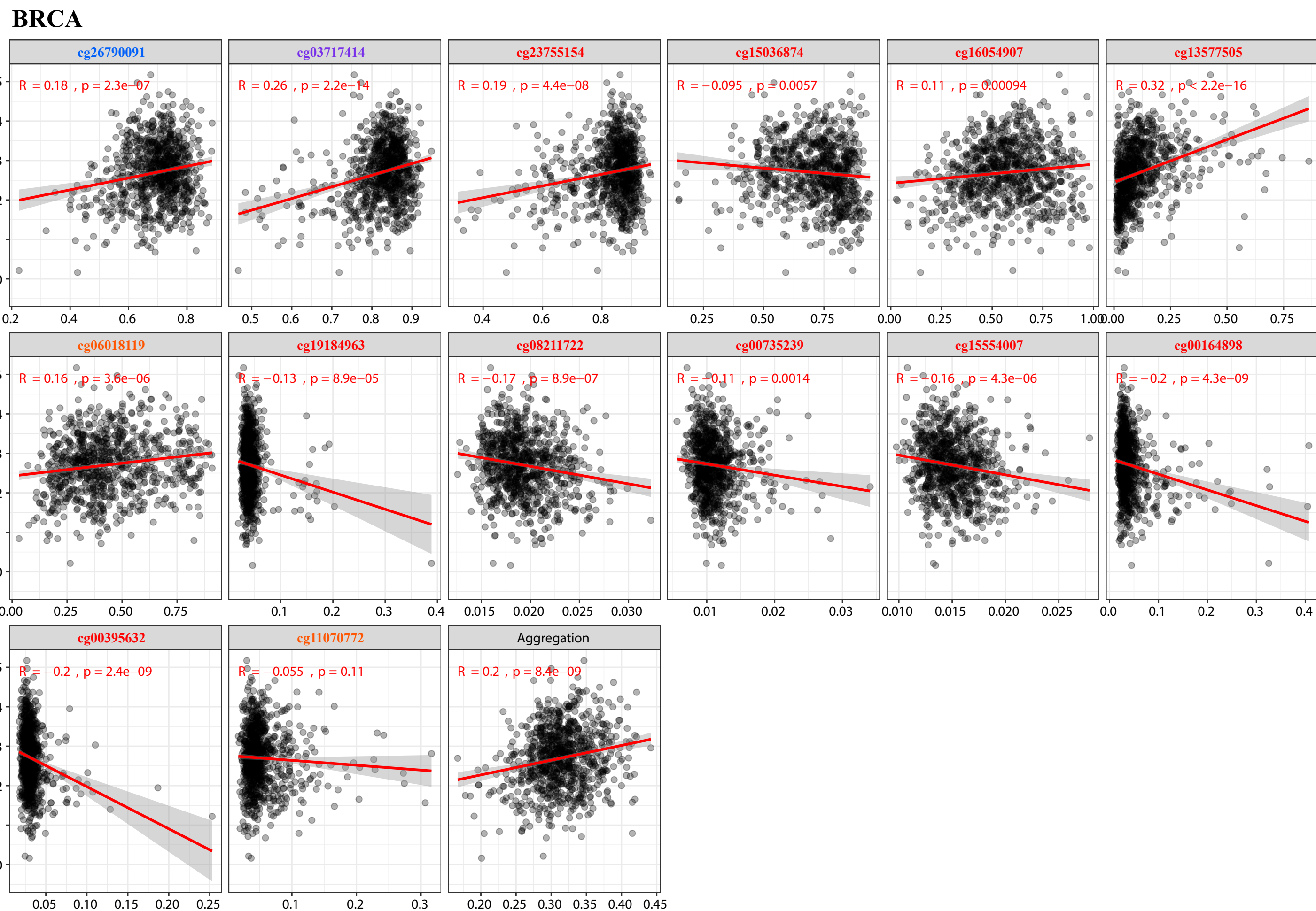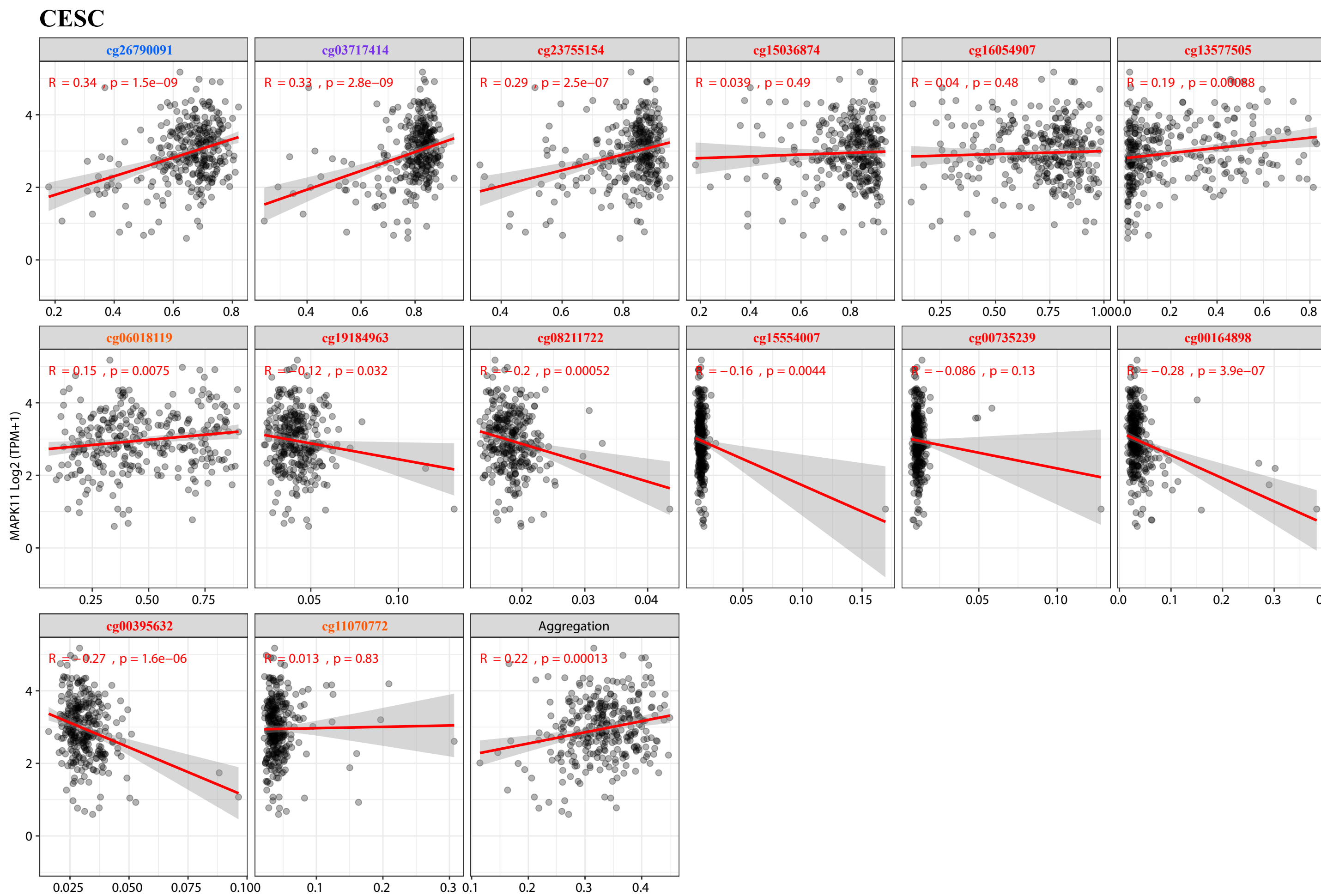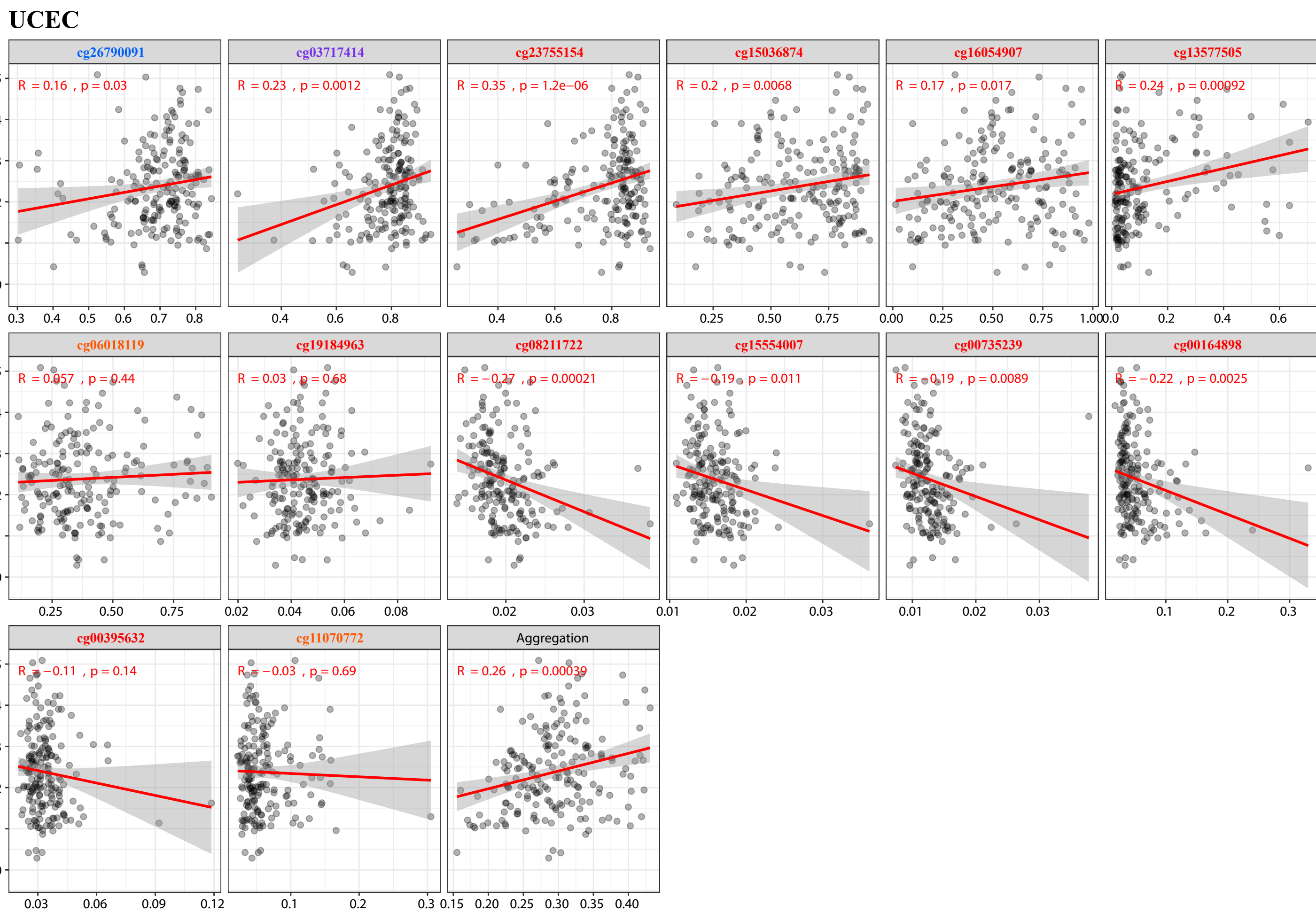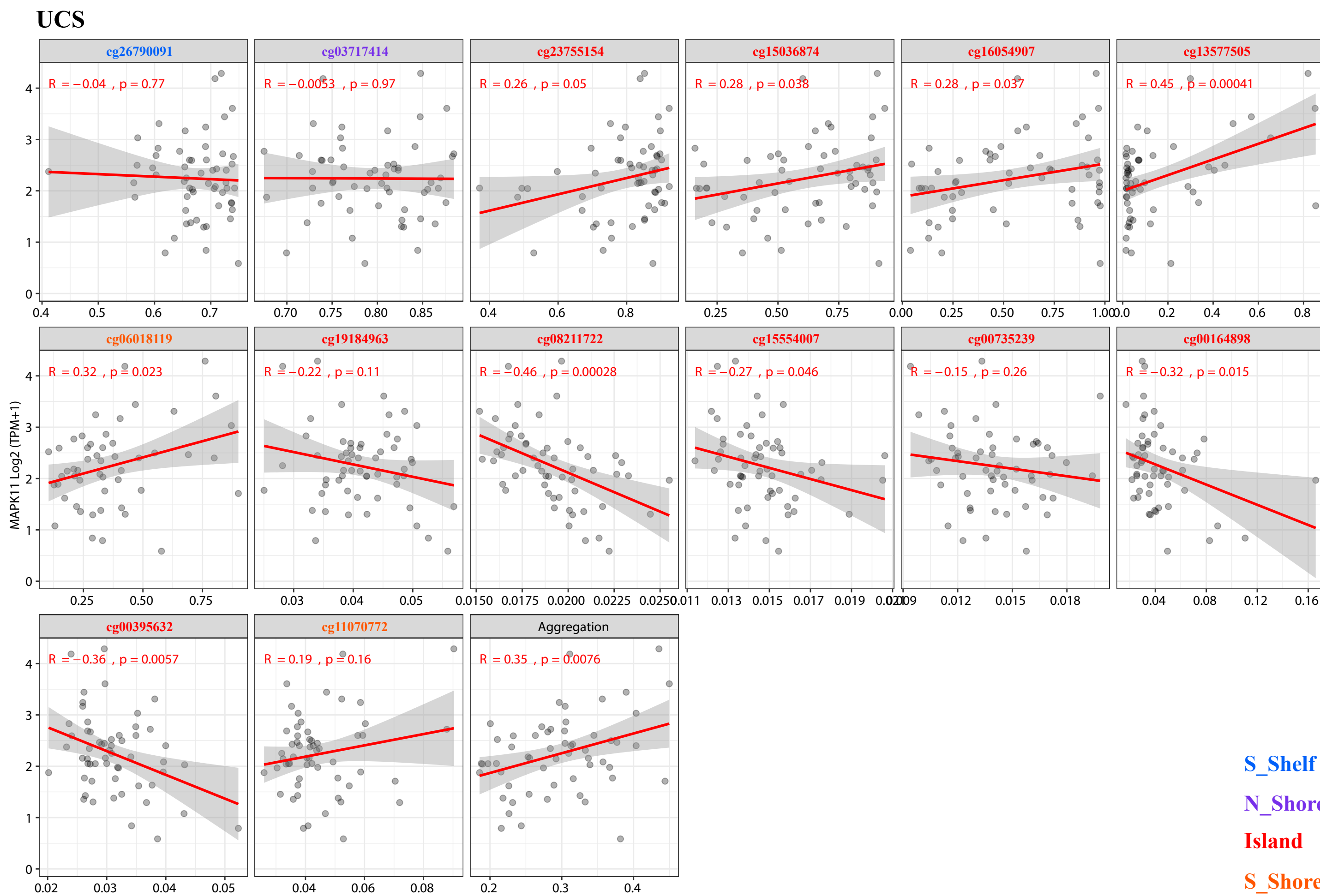

S\_Shelf  
N\_Shore  
Island  
S\_Shore

Supplement: Supplementary file 4 — Additional file 4:. MAPK11 Methylation and correlation of expression with methylation in BRCA, CESC, UCEC and UCS of all the 14 probes. [file 13048_2021_834_MOESM4_ESM.pdf]

BRCA

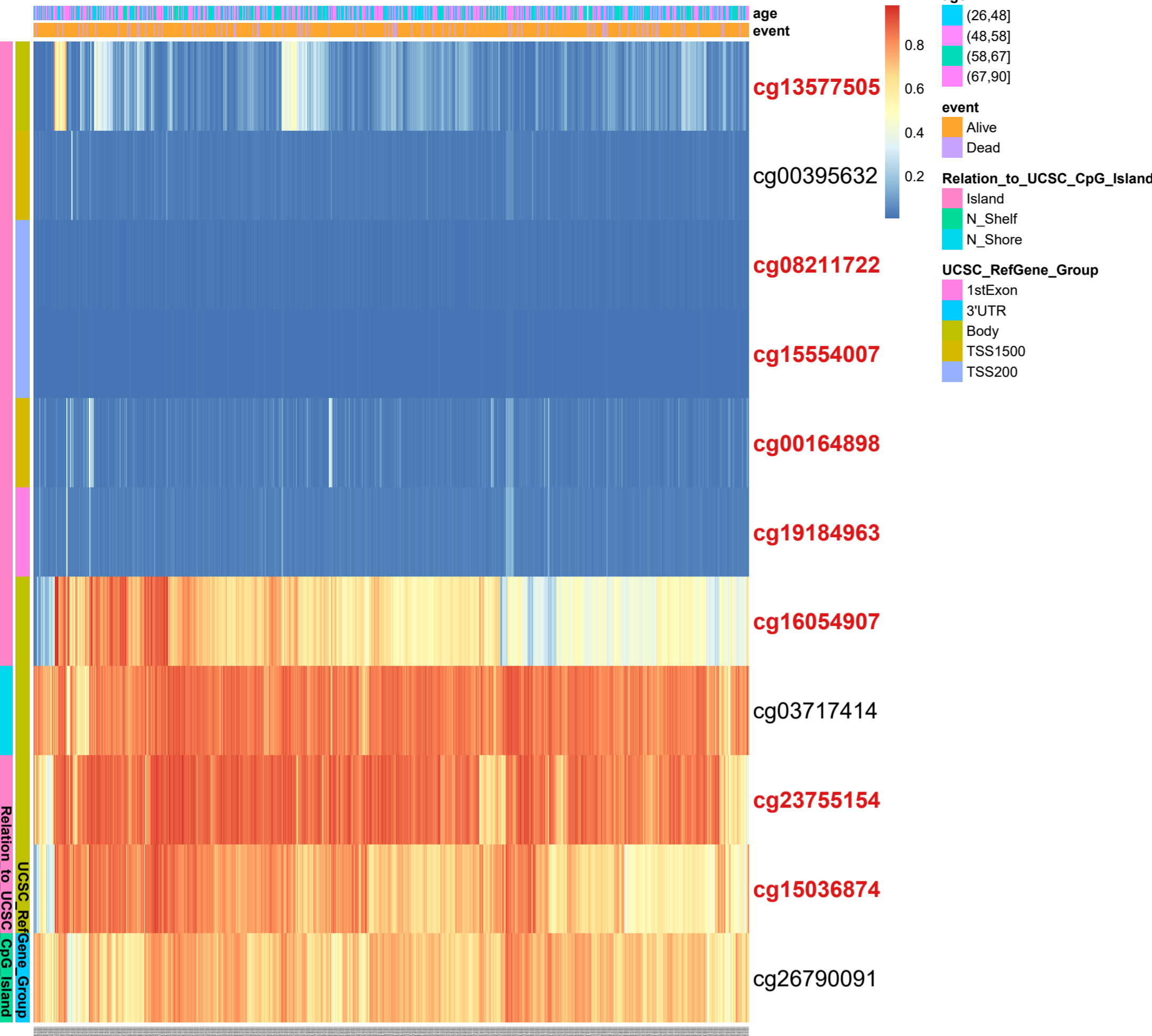

CESC

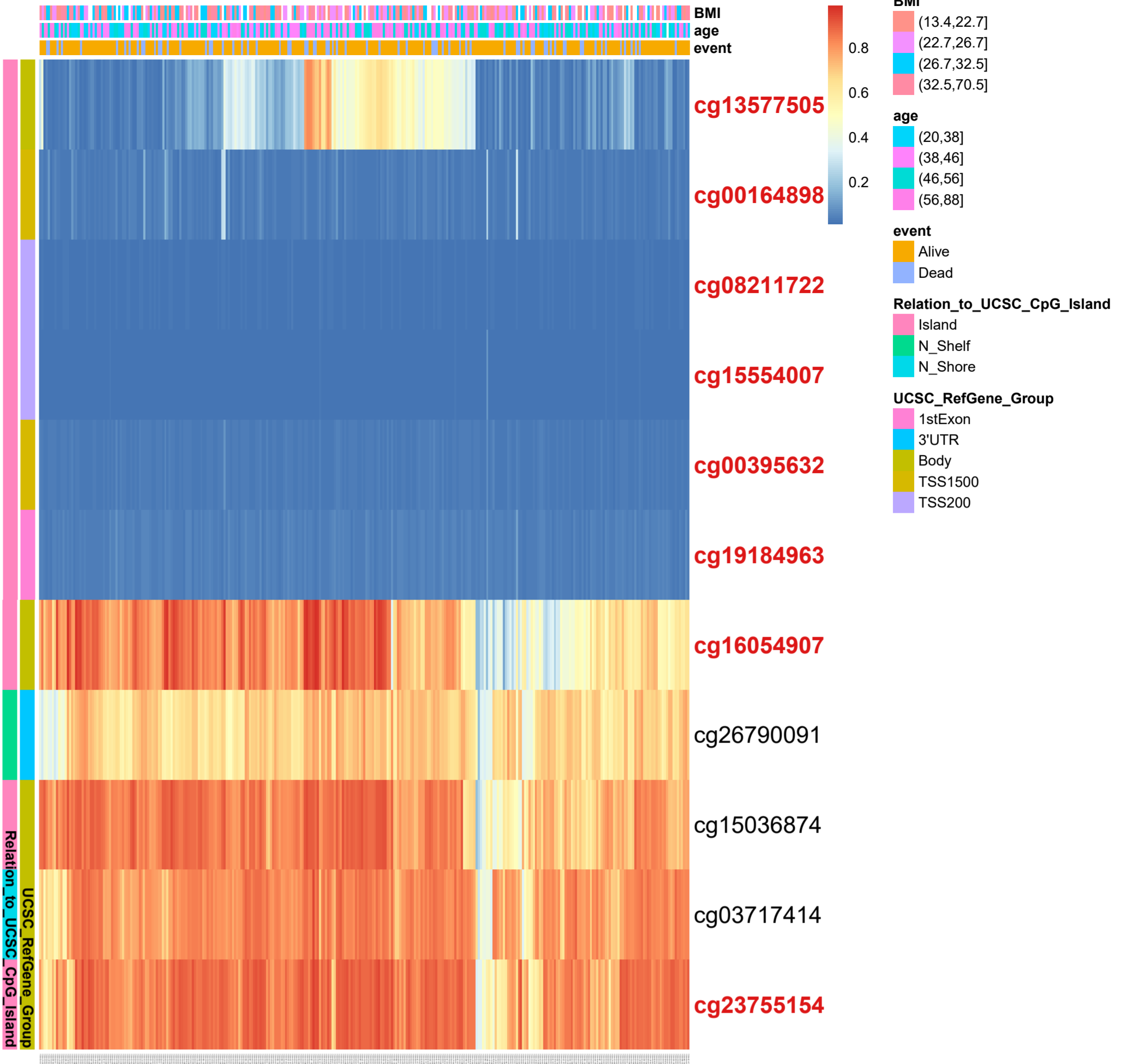

UCEC

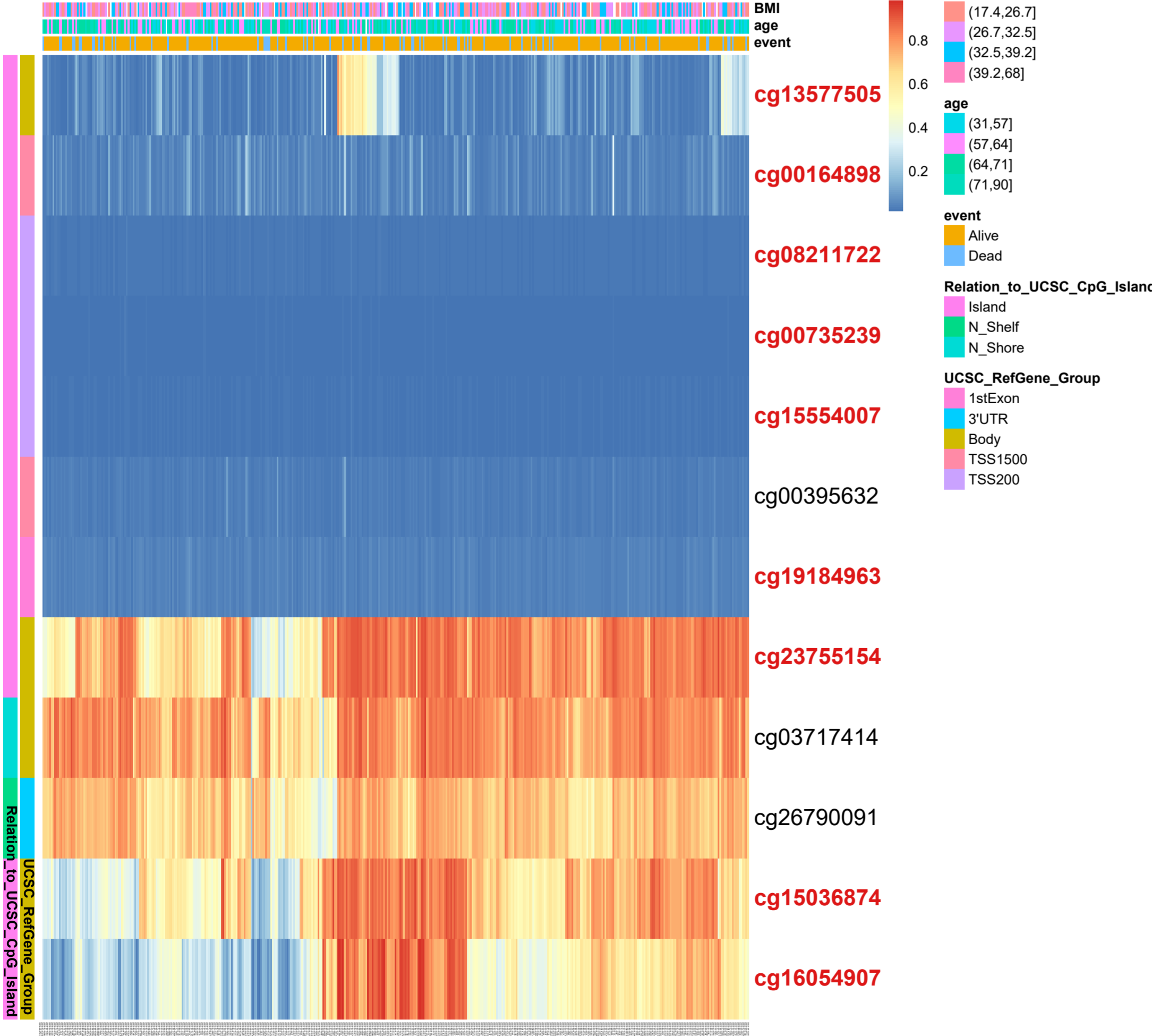

UCS

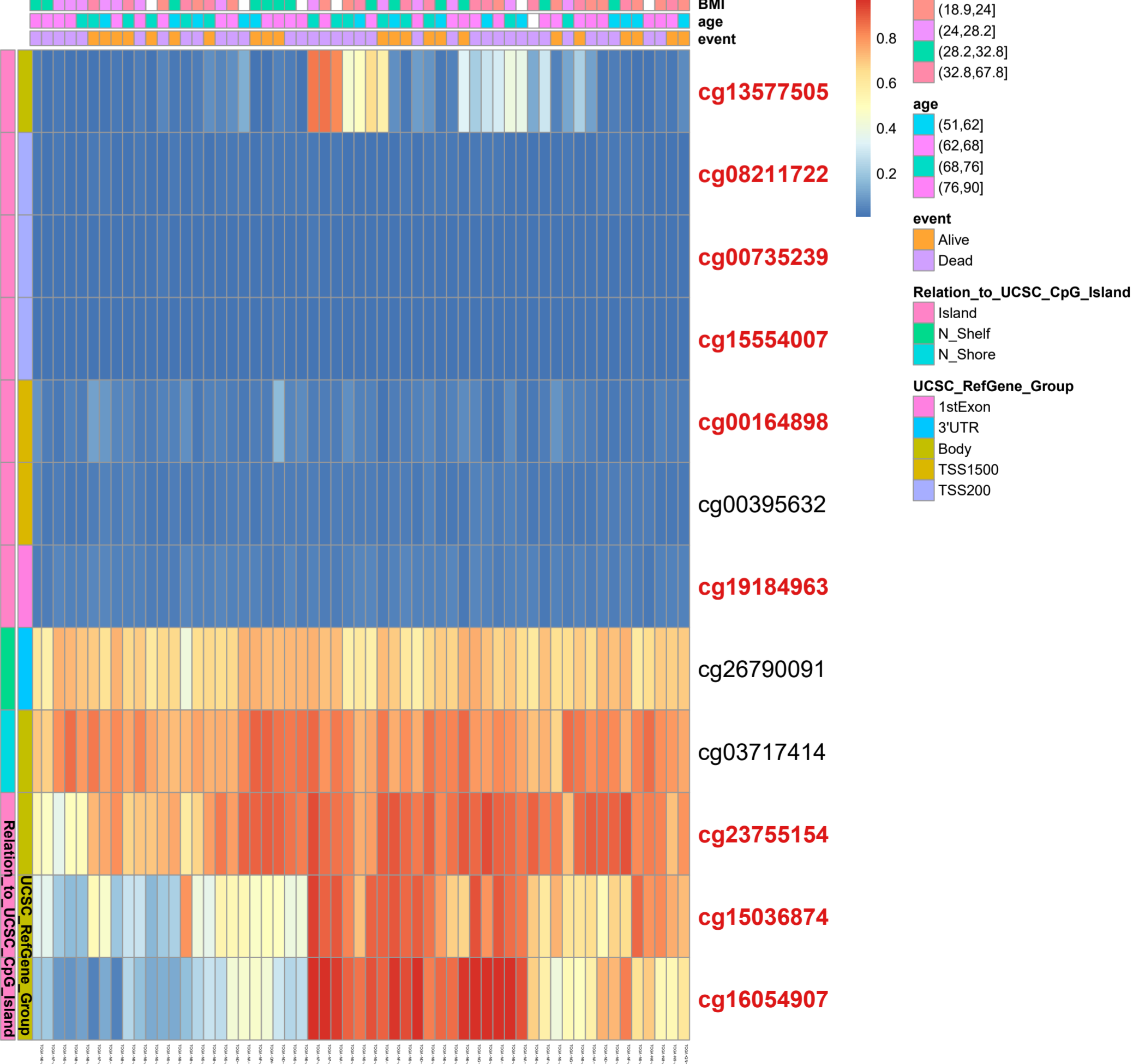

Supplement: Supplementary file 6 — Additional file 6:. Heatmap of all 14 methylated MAPK11 probes in BRCA, CESC, UCEC and UCS. The CpG island probes are presented in red. [file 13048_2021_834_MOESM6_ESM.pdf]
