# Supplementary material for: p38β - MAPK11 and its role in female cancers
Source: J Ovarian Res. 2021 Jun 26;14:84. doi: 10.1186/s13048-021-00834-9 (PMC8236201; doi:10.1186/s13048-021-00834-9)
Supplement: Supplementary file 5 — Additional file 5:. Detailed presentation of methylation status of the MAPK11 gene in BRCA, CESC, UCEC and UCS as retrieved from MEEXPRESS analysis. The CpG island probes are presented in red and asterisks annotations represent statistical significance. [file 13048_2021_834_MOESM5_ESM.pdf]

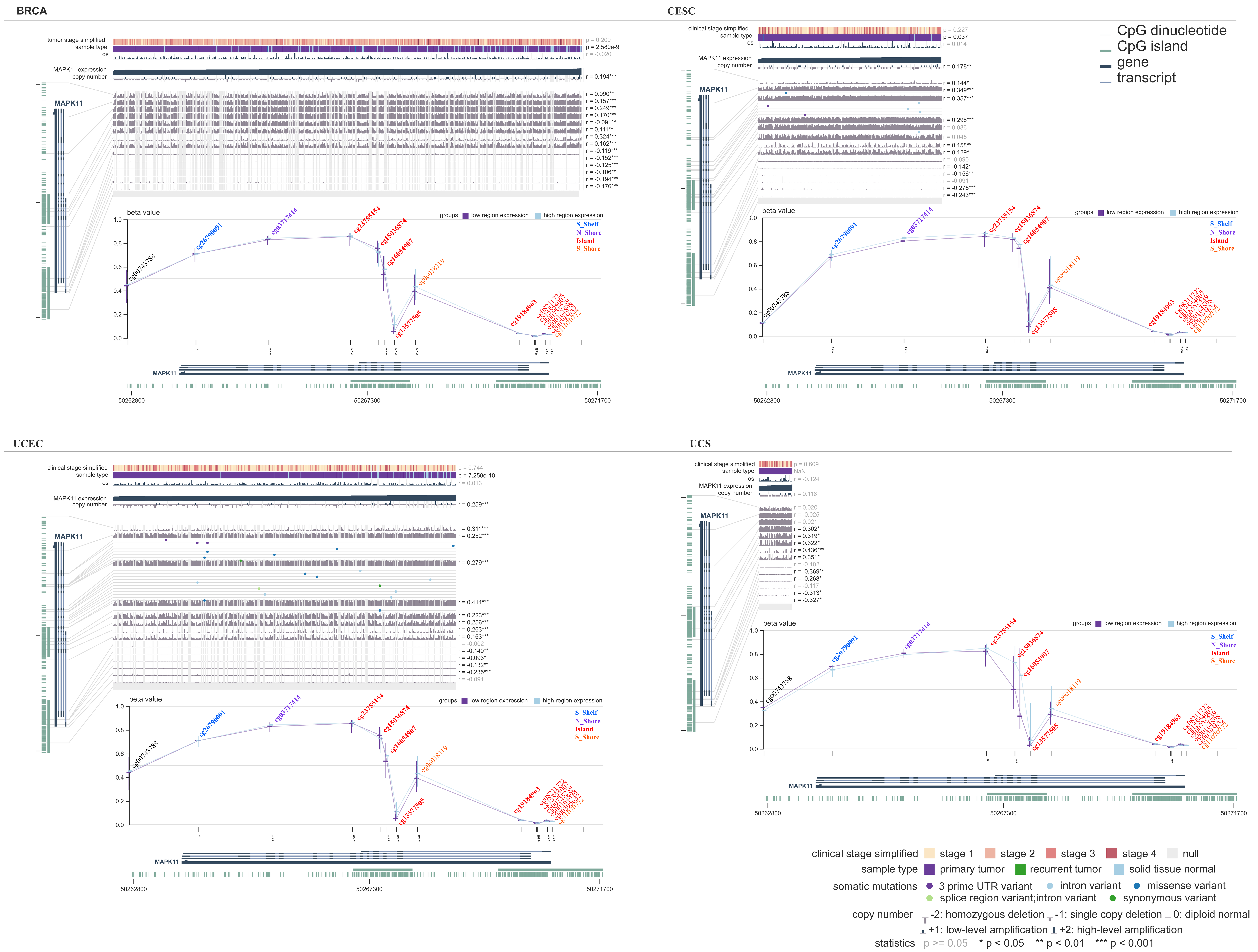

BRCA
tumor stage simplified
sample type
os
MAPK11 expression
copy number
p = 0.200
p = 2.580e-9
r = -0.020
r = 0.194***
MAPK11
r = 0.090**
r = 0.157***
r = 0.249***
r = 0.170***
r = -0.091**
r = 0.111**
r = 0.324***
r = 0.162***
r = -0.119***
r = -0.152***
r = -0.125***
r = -0.106**
r = -0.194***
r = -0.176***
beta value
groups
low region expression
high region expression
S_Shelf
N_Shore
Island
S_Shore
cg00743788
cg26790091
cg03717414
cg23755154
cg15036874
cg16054907
cg06018119
cg13577505
cg19184963
50262800
50267300
50271700
CESC
clinical stage simplified
p = 0.227
p = 0.037
r = 0.014
r = 0.178**
r = 0.144*
r = 0.349***
r = 0.357***
r = 0.298***
r = 0.086
r = 0.045
r = 0.158**
r = 0.129*
r = -0.090
r = -0.142*
r = -0.156**
r = -0.091
r = -0.275***
r = -0.243***
CpG dinucleotide
CpG island
gene
transcript
UCEC
p = 0.744
p = 7.258e-10
r = 0.013
r = 0.259***
r = 0.311***
r = 0.252***
r = 0.279***
r = 0.414***
r = 0.223***
r = 0.256***
r = 0.263***
r = 0.163***
r = -0.002
r = -0.140**
r = -0.093*
r = -0.132**
r = -0.235***
r = -0.091
UCS
p = 0.609
NaN
r = -0.124
r = 0.118
r = 0.020
r = -0.025
r = 0.021
r = 0.302*
r = 0.319*
r = 0.322*
r = 0.436***
r = 0.351*
r = -0.102
r = -0.369**
r = -0.268*
r = -0.117
r = -0.313*
r = -0.327*
clinical stage simplified
stage 1
stage 2
stage 3
stage 4
null
sample type
primary tumor
recurrent tumor
solid tissue normal
somatic mutations
3 prime UTR variant
intron variant
missense variant
splice region variant;intron variant
synonymous variant
copy number
-2: homozygous deletion
-1: single copy deletion
0: diploid normal
+1: low-level amplification
+2: high-level amplification
statistics
p >= 0.05
* p < 0.05
** p < 0.01
*** p < 0.001
